# Coercive mating has no impact on spatial learning, cognitive flexibility, and fecundity in female porthole livebearers (*Poeciliopsis gracilis*)

**DOI:** 10.1111/jfb.15696

**Published:** 2024-02-25

**Authors:** Tiffany R. Ernst, R. M. H. W. Hogers, A. Korosi, J. L. van Leeuwen, A. Kotrschal, Bart J. A. Pollux

**Affiliations:** ^1^ Department of Animal Sciences, Experimental Zoology Group Wageningen University Wageningen The Netherlands; ^2^ University of Amsterdam, Swammerdam Institute of Life Sciences, Center for Neuroscience, Brain Plasticity Group Amsterdam The Netherlands; ^3^ Department of Animal Sciences, Behavioral Ecology Group Wageningen University Wageningen The Netherlands

**Keywords:** coercive mating, Poeciliidae, pregnancy, reversal learning, spatial learning

## Abstract

Coercive mating is a sexual selection strategy that is likely to influence female cognition. Female harassment levels have been linked to altered brain gene expression patterns and brain size evolution, suggesting females may respond to coercive mating by investing energy into “outsmarting” males. However, females exposed to coercive males have decreased foraging efficiency and likely increased stress levels, suggesting their brain function might instead be impaired. While it is therefore likely that coercive mating impacts female cognitive abilities, a direct test of this idea is currently lacking. In this study, we investigate the impact of coercive mating on female spatial memory and cognitive flexibility in a species with prevalent coercive mating. We compared the performance of female porthole livebearers (*Poeciliopsis gracilis*), which had been previously housed alone or with a coercive male, in both a spatial food localization task and a reversal learning task. While we found that both single and paired fish exhibited high proficiency in learning both tasks, we found no differences in learning ability between females that had or had not experienced coercive mating. In addition, our study found that the presence of a coercive male had no impact on female fecundity, but did influence female mass and standard length. Several studies have assumed that the presence of males, particularly coercive males, may affect the cognitive performance of female fish. However, our study shows that for some species females adapted to coercive mating regimes may be unaffected by male presence with regards to some cognitive tasks.

## 1 INTRODUCTION

Sexual conflict is a major driver of the co‐evolution between sexes and occurs when males and females have different fitness optima for a shared interaction or activity (Chapman et al., 2003; Parker, 2006; Pischedda & Stewart, 2016). For many animals that reproduce sexually, copulation and/or reproduction is this “shared interaction or activity” that sets the battleground for both pre‐ and post‐copulatory sexual conflicts.

Specifically, pre‐copulatory sexual conflicts often occur as a result of secondary sexual characteristics or mating behaviors that influence which individuals mate and thereby which traits are passed onto their offspring, and these strategies can be broadly categorized by the degree of cooperation between mating individuals. For example, courtship behaviors or attractive sexual characteristics (e.g., vibrant coloration, ornamentation, or exaggerated body forms) displayed by one sex aim to earn the cooperation of the other, either by earning the right to mate by outcompeting members of the same sex or earning the approval of the opposite sex (Andersson & Iwasa, 1996; Parker, 2006). Conversely, other species adopt sexual characteristics or behaviors that promote non‐cooperative or coercive mating, a strategy which bypasses the need for cooperation by circumventing the mate selection of one sex in favor of the other (Andersson & Iwasa, 1996; Parker, 2006). Coercive mating thus offers an additional sexual selection strategy that favors the coercer and hinders the coerced (Bisazza et al., 2001; Clutton‐Brock & Parker, 1995). Coercive mating is prevalent across the animal kingdom and can include a wide variety of aggressive tactics, including sexual harassment, forced copulation, and intimidation or punishment (as reviewed by Clutton‐Brock and Parker (1995)). For species which rely on coercive mating, the coercive sex is motivated to copulate frequently to improve their reproductive success, while the coerced sex evolves strategies to avoid copulation unless absolutely necessary, creating a behavioral “arms race” between the sexes (Parker, 2006).

Live‐bearing fishes from the family Poeciliidae have been long‐studied models to investigate how sexual conflict influences both sexual selection and the evolution of different reproductive strategies (Cummings, 2018; Furness et al., 2019). Fish species from this family exhibit a wide variety of mating tactics, including species where males perform courtship behaviors, species where males only copulate through coercive mating, and species where males show a mix of both strategies (Farr, 1989; Pollux et al., 2014). The majority of poeciliid species mate exclusively through a form of coercive mating, also known as sneaky copulation, where males approach a female from behind and thrust their gonopodium (a modified anal fin) into the gonoduct of the female to inseminate her, seemingly without consent or cooperation (Bisazza & Pilastro, 1997; Evans et al., 2011; Farr, 1989; Pilastro et al., 1997). These coercive mating attempts can reach rates of up to one mating attempt per male per minute in some poeciliid species (Bisazza & Marin, 1995; Houde, 1997; Pilastro et al., 2003). Due to the prevalence and persistence of these mating attempts, researchers have postulated that coercive mating is likely detrimental to females in these environments. Females have to endure near‐constant (often unsuccessful; Bisazza & Marin, 1995) mating attempts, and they lose much of their selective power during copulation. In addition, coercive mating decreases female foraging (Griffiths, 1996; Magurran & Seghers, 1994a; Pilastro et al., 2003) and feeding efficiency (Schlupp et al., 2001), much of which has been attributed to the changes in behavior females exhibit in the presence of coercive males (Magurran & Seghers, 1994a). Consequently, this shift in behavior often leaves females more vulnerable to predation (Magurran & Seghers, 1994b). Taken together, we see that coercive mating has obvious drawbacks for females, both directly, as females change behavior and spend energy avoiding male mating attempts, and indirectly, as females lose the majority of their pre‐copulatory sexual selective power.

Therefore, in poeciliid species with exclusively coercive mating, females have had to adapt to near‐constant mating attempts by adopting their own anti‐mating tactics. For example, females are highly evasive and most coercive mating attempts are unsuccessful (Bisazza & Marin, 1995). Virgin females or females who have been deprived of males are more cooperative during coercive mating attempts, indicating that females can choose to cooperate with certain males when they need new sperm (Bisazza et al., 2001). Females can also aggregate together to decrease the male focus on a single female while also increasing their collective foraging efficiency (Pilastro et al., 2003), counteracting some of the detriments to foraging efficiency brought on by female evasive maneuvers. Many poeciliid species have also evolved the ability to store sperm, which may be a female strategy that allows them to avoid the company of males once they have mated (Constantz, 1984). In addition to changes in behavior and physiology, poeciliid females also experience changes in the brain as a result of coercive mating. Females exposed to coercive mating show a negative association between mate‐preference genes and their interaction with coercive males, while females exposed to courting males show a positive association, emphasizing that this differential gene regulation is due to male behavior rather than just presence (Cummings, 2012; Lynch et al., 2012). In addition, female brain size increases over generations in environments with males with longer gonodopodia, further suggesting that female cognition is modulated by coercive mating (Buechel et al., 2016).

Despite ample evidence that coercive mating impacts the female brain and behavior, a direct test of whether female cognitive abilities are impacted by coercive matings—and whether these neurological changes may persist after coercive males are no longer present—is currently lacking. Here we perform such a test in female porthole livebearers (*Poeciliopsis gracilis*) focused on female food localization and cognitive flexibility. We exposed females to 75 days of either coercive mating or sexual seclusion and subsequently investigated their performance in a spatial and a reversal learning task. We chose spatial learning because of the general importance of learning about and remembering spatial characteristics in the home range of such a resident species in small freshwater bodies. After the spatial memory task, we assessed females in a reversal learning task to determine if exposure to coercive mating had any effects on cognitive flexibility. We hypothesize that females will cognitively adjust in response to the coercive mating environment in one of two ways. First, females exposed to coercive mating will be better at learning the location of the food reward compared to isolated females. Moreover, if coercive mating is positively correlated with phenotypic plasticity in the brain, then females exposed to coercive mating may also have greater cognitive flexibility to learn the new location of the food reward after it has changed. Conversely, females exposed to coercive mating will be worse at finding the food reward in both the spatial and reversal learning tasks because of either an energetic trade‐off in which they have diverted energy towards better evasive behaviors or post‐copulatory mate‐choice strategies, and/or due to a generally negative impact of stress on cognitive abilities (Salena et al., 2021). By studying female cognition after exposure to coercive mating and not while males are present, our study aims to disentangle whether the previously reported changes in female behavior in coercive mating environments are simply a female's behavioral response to male presence or an indication of more long‐term changes in female cognition as a result of coercive mating.

## 2 METHODS AND MATERIALS

**FIGURE 1:**
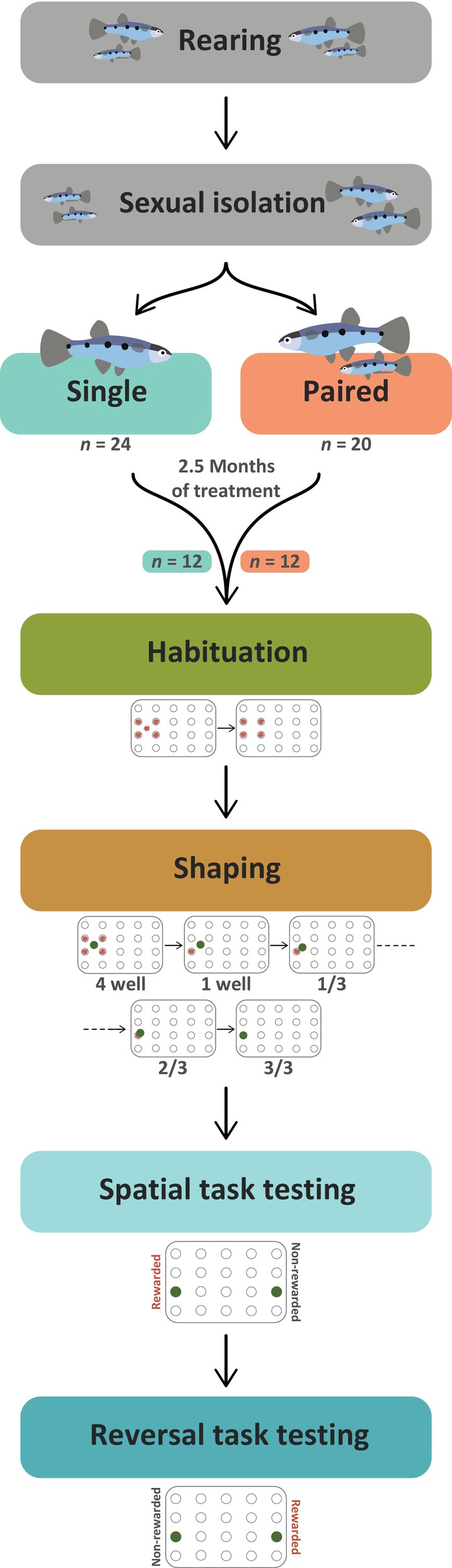
A schematic overview of all experiments, including all preparatory, training, and testing phases.

Our experimental protocol for assessing female cognition in response to coercive mating consisted of several preparatory and training phases prior to behavioral testing, outlined broadly in Figure 1 and described in detail in the sections below.

Briefly, all *P. gracilis* used in this study were reared in community tanks where they were allowed to freely copulate before they were separated by sex at approximately 5 months old. This period of sexual isolation lasted approximately 2.5 months before fish were divided into two treatments: single or paired (labeled S# and P#, respectively), where single fish remained sexually isolated and paired fish were housed with a coercive male. All fish then experienced this treatment for another 2.5 months before 24 fish were selected (12 single and 12 paired) for the experiment and moved into the experimental tanks for behavioral training and testing. Behavioral training consisted of a short period of habituation before a longer period of shaping, where fish were allowed to learn at their own pace. After the fish reached the pre‐determined learning criteria, they proceeded to behavioral testing, where they were assessed first in a spatial learning task, followed directly by a reversal learning task.

### 2.1 Study species

*P. gracilis* (Heckel, 1848) is a freshwater fish native to river basins along the Atlantic (Gulf) slope of Mexico, particularly in the Mexican states of Veracruz and Oaxaca (Miller et al., 2005). However, outside of their native range, *P. gracilis* has been introduced and has established invasive populations in several river basins in central Mexico and along the Pacific slope of Mexico into Honduras (Campuzano‐Caballero & Uribe, 2017; Meja‐Mojica et al., 2012). The fish used in our experiment were laboratory‐born individuals derived from laboratory stocks originally collected from a small tributary of the Rio Motagua, near the village Jones in Zacapa, Guatemala (2003/2004). Invasive populations of *P. gracilis* have been shown to compete with other local foraging native fishes, making them a threat to these river ecosystems (Camacho‐Cervantes et al., 2019). Previous research investigating the invasive success of *P. gracilis* has found that females are particularly bold—compared to males and unlike other invasive poeciliids like the more commonly studied *Poecilia reticulata*—allowing them to easily disperse into and thrive in new environments (Aceves‐Fonseca et al., 2022; Harris et al., 2010; Kim, 2014). In addition, habitat complexity in these environments had no effect on the females' ability to find food (Aceves‐Fonseca et al., 2022). Given the success of *P. gracilis* in establishing successful and often invasive populations in a variety of river ecosystems (Miller et al., 2005), we hypothesized that spatial learning for food localization, coupled with cognitive flexibility to adapt once dispersed, would be particularly advantageous for females of this species.

### 2.2 Experimental animals

*P. gracilis* used in this study were reared in 30‐L tanks (density ~1 fish/L) with constant aeration and a water temperature of 25 ± 2°C under a 12 h light/12 h dark cycle in the Live‐Bearing Fish Facility of CARUS (Wageningen University & Research; Wageningen, The Netherlands). Prior to experiments, fish were fed a high‐food diet consisting of 0.15–0.2 g of 200–300 μm sterilized CAVIAR (BernAqua) and ~8 mg nauplii *Artemia spec*. (Salt Lake Aquafeed, Premium Artemia Cysts, hatched on‐site in CARUS) daily at 8:00 and 16:00, respectively. All tanks were monitored daily for fish welfare and cleanliness; offspring were also collected when necessary. All the *P. gracilis* used in this study were used under approval by the animal ethics committee of Wageningen University & Research (2020.W‐0027).

**FIGURE 2:**
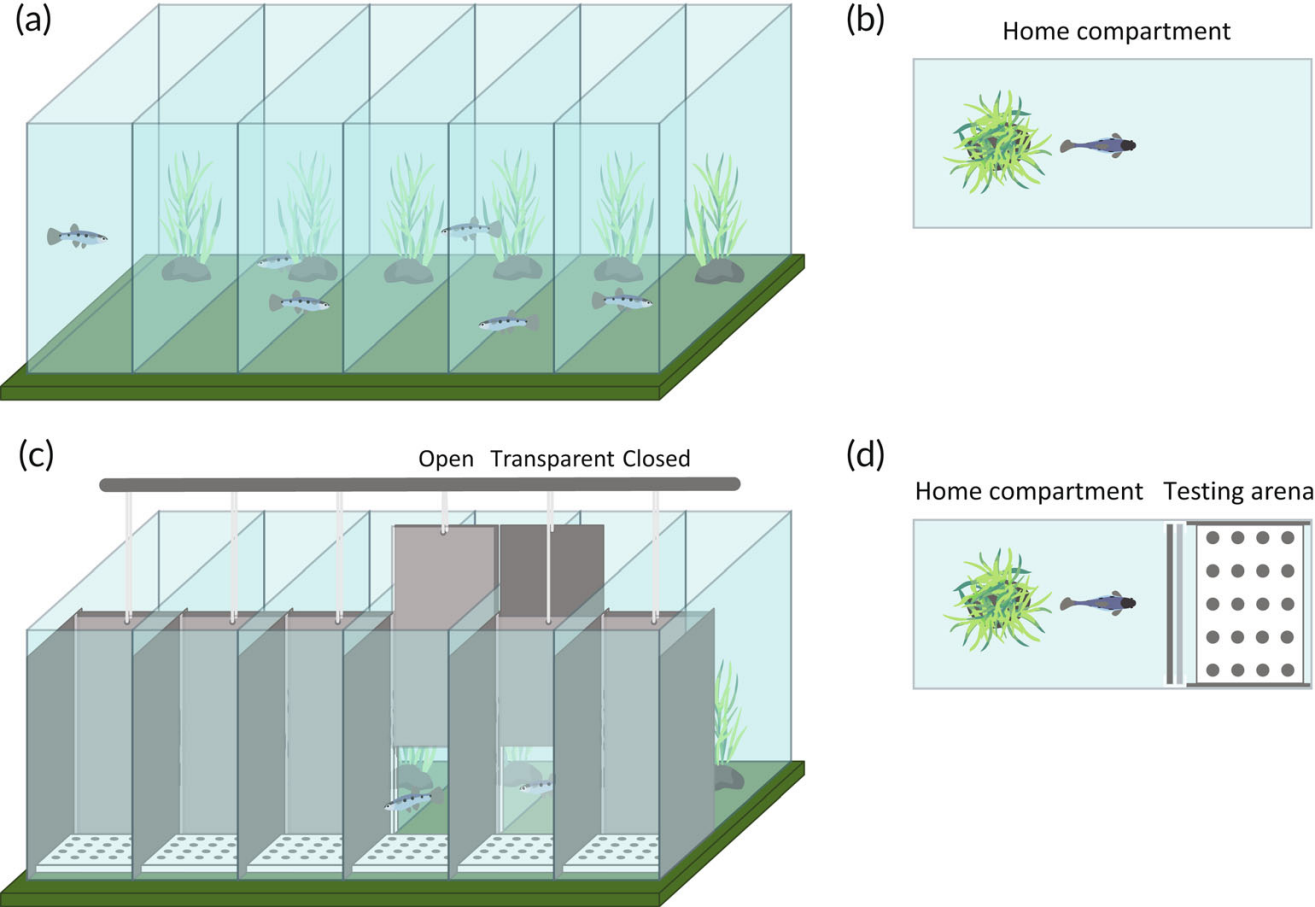
Schematic of the tanks where fish were housed before and during behavioral trials. (a) A three‐dimensional depiction of the home tanks where fish were housed prior to behavioral testing. (b) A lateral‐view schematic of each home tank. (c) A three‐dimensional depiction of the experimental tanks where fish were housed during behavioral testing. The guillotine doors (opaque and transparent) in these tanks could be open, partially open (where the opaque door is open but the fish can look through the closed transparent door), or closed. (d) A lateral‐view schematic of each experimental tank, indicating the home compartment and the testing arena.

Fish were allowed to breed freely after sexual maturation. At approximately 5 months of age, all the male fish were removed from each tank and housed in an adjacent tank. Females were then left isolated from male contact for 2.5 months, during which time offspring were collected from the tanks when necessary. After the 2.5 months of sexual isolation, 44 females (single, 24; paired, 20) were randomly selected and rehoused in 10‐L home tanks. Each home tank consisted of a 14 × 37 × 29 (width × depth × height) cm, 13‐L compartment within a larger six‐compartment tank, as shown in Figure 2a,b.

Single fish were housed individually to prevent male contact and thus coercive mating, while paired fish were housed with a randomly selected male to allow for mate contact and coercive mating. This compartment structure allowed fish to see and interact with at least one neighboring fish (fish housed in compartments on the end of each tank had access to only one neighbor) to reduce isolation stress, particularly in single females. Each tank also contained a plastic aquarium plant for fish to use as shelter and enrichment. Fish in home tanks were now given an additional meal on weekdays (at ~12:00) to prepare them for experiments: 2–3 mL of high‐density adult *Artemia* mixed with garlic (Aquadip #0355, defrosted in tank water prior to administration). The garlic in the food was selected to increase fish motivation for the food source by creating a strong olfactory cue. Each home tank was monitored for fish welfare and offspring production for 1.5 months.

Fish were selected for the experiment based on their offspring production during the monitoring period. The average number of offspring per brood was calculated for each fish at the end of the monitoring period. Fish were subsequently divided into ranks within their treatment group (single or paired), ranks 1–7 for single fish and 1–6 for paired fish, where each rank represented a range of average offspring numbers. For example, rank 1 (for both groups) included fish which had less than one offspring per brood during the monitoring period, while rank 2 included fish which had one up until two offspring per brood on average. Fish were then randomly selected from each rank within their group until 12 individuals were chosen from each group (refer to Figure S1 for a visualization of this selection). Selecting the fish in this way allowed us to randomly select individuals from each group while also ensuring that we selected fish from across the distribution of offspring production within each treatment since reproductive output may have an influence on fish behavior. To indirectly check whether the addition of a male, or the food regime of the behavioral experiments, had an impact on female food consumption, we monitored their offspring production throughout the remainder of the experiment. This is based on previous work by Reznick et al. (1996) which found that poeciliid females exposed to food restrictive diets produce less offspring per brood, focusing instead on larger offspring with larger fat reserves.

Selected fish were moved individually to one of the home compartments of the experimental tanks shown in Figure 2c,d. The males from the paired fish were moved to a community tank of males outside of the behavioral set‐up.

### 2.3 Experimental tanks

Experimental tanks were identical in size and shape to the home tanks but were modified to accommodate behavioral testing. The front 13 cm (depth) of each compartment was lined with opaque gray panels to prevent the fish from seeing into neighboring compartments during the behavioral testing and learning from watching their neighbors (Brown & Laland, 2003). Each tank was equipped with a pair of guillotine doors, one opaque and one translucent, to separate the home compartment from the behavioral arena (Figure 2c,d). During testing each guillotine door could be moved independently, as indicated in Figure 2c. After the fish had acclimated to the behavioral tanks (~1 week) the doors were slid into the slots and the white wells‐plate for behavioral testing was placed in the front of the tank to establish the testing arena. Once the doors were inserted, fish were confined to the home compartments outside of the designated testing periods.

### 2.4 Behavioral Training and Testing

During training, fish were no longer fed high‐food diets and instead only received food during the designated training periods or during normal feeding periods at the weekends. During testing (spatial and reversal) the fish performed behavioral trials for 12 consecutive days and only received food during the behavioral trials; this meant that fish which did not solve the task during the allotted 15 min per trial were kept on minor food restriction outside of the testing periods to increase appetite and participation. Both training and testing were performed twice a day during designated training periods: morning (~9:00–10:30) and afternoon (~15:00–16:30). These times were selected to replace the normal feeding times of the fish.

All behavioral training and testing was observed and scored manually by one experimenter and recorded using a pair of GoPro Hero 11 cameras. For trials in which the experimenter missed a score or was otherwise unsure of the scoring, the video recordings were checked to confirm the scores. During all trials (both training and testing), the amount of food fish ate during the trial was also recorded on a scale from 0 to 3, where 0 meant none eaten, 1 meant a small amount eaten (≤1 *Artemia*), 2 meant around half of the *Artemia* was eaten, and 3 meant all of the *Artemia* were eaten. Training consisted of two main phases: habituation and shaping. This training period was followed by the task testing for spatial learning and then the test for reversal learning. These four phases of behavioral assessment are described below in more detail and visualized in Figure 1.

#### 2.4.1 Habituation

To reduce stress and enhance learning, fish were given a period of habituation as part of their training to acclimatize them to the wells‐plate and the moving doors. After being placed in their behavioral tanks, fish were fed ~12–16 *Artemia* in the water above the wells‐plate. During pre‐habituation, the guillotine doors were not positioned in the slots, as described above, instead they were only added once fish began habituation. After this first training period of pre‐habituation, the fish were trained to eat from the wells‐plate; at the start of each training period three or four *Artemia* were pipetted into four adjacent wells and onto the plate in the middle of the four wells, as indicated by the wells‐plate visualized in Figure 1. The fish were left to eat the *Artemia* until the next training period. Before each new training period the wells‐plate was emptied of any remaining *Artemia* before being refilled for the new trial. If the fish successfully ate the *Artemia* before the next training period, the *Artemia* was only administered inside the wells and not on top of the plate in the next training period (habituation; Figure 1). All fish performed a total of 12 habituation trials (6 days of training, two trials per day).

#### 2.4.2 Shaping

During the shaping phase, the fish had to learn to associate the location of a green disk with the location of the *Artemia*. Before the start of the first training period, the opaque and transparent doors were slotted into place to separate the home compartment from the testing arena. Each training period consisted of three consecutive trials which each lasted a maximum of 15 min. At the start of each training period the wells‐plate was cleaned if necessary and 12–16 *Artemia* were divided evenly between four adjacent wells. A plastic green disk was then positioned in the middle of these four wells (4 well phase; Figure 1). To start each trial, the opaque door was first opened for ~10 s to allow the fish to inspect the testing arena. The transparent door was then opened to allow the fish to enter the training arena and eat the *Artemia*. Fish were scored successfully if they ate any of the *Artemia* within 5 min. If the fish did not eat within 5 min, the doors were left open until the fish had eaten from the wells‐plate or for a maximum of an additional 10 min. If the fish successfully ate within 5 min for nine out of 12 consecutive trials, in the next trial three or four *Artemia* were only administered to one well (1 well phase; Figure 1). In the 1 well phase, fish had to successfully eat from the well in the first 5 min of nine out of 12 consecutive trials. Again, if the fish did not eat in the first 5 min the doors were left open until they ate, or for a maximum of 10 additional minutes.

After the 1 well phase, fish were moved into the 1/3 phase; in this phase, the food reward was left in the same well as in the 1 well phase but the disk was moved so that the edge of the disk was touching the edge of the well (1/3 phase; Figure 1). Again, fish had to succeed within 5 min in nine out of 12 consecutive trials to move forward and were given extra time in each trial if needed. From the 1/3 phase, fish proceeded to the 2/3 phase, where the disk was moved so that it covered half of the well containing the food reward (2/3 phase; Figure 1). Fish only had to succeed in one trial at this coverage level to proceed to the next phase. The final phase of shaping was the 3/3 phase, where the disk was moved so that it completely covered the well containing the food reward (3/3 phase; Figure 1). In this phase, fish had to push the disk out of the way to access the food reward; fish were considered to have learned the association between the green disk and the food reward if they could push the disk to get the food in the first 5 min in nine out of 12 consecutive trials. Again, fish were given up to 10 min extra time if needed. Once fish had passed the criteria needed to complete the 3/3 phase, they could move to the spatial learning task.

**FIGURE 3:**
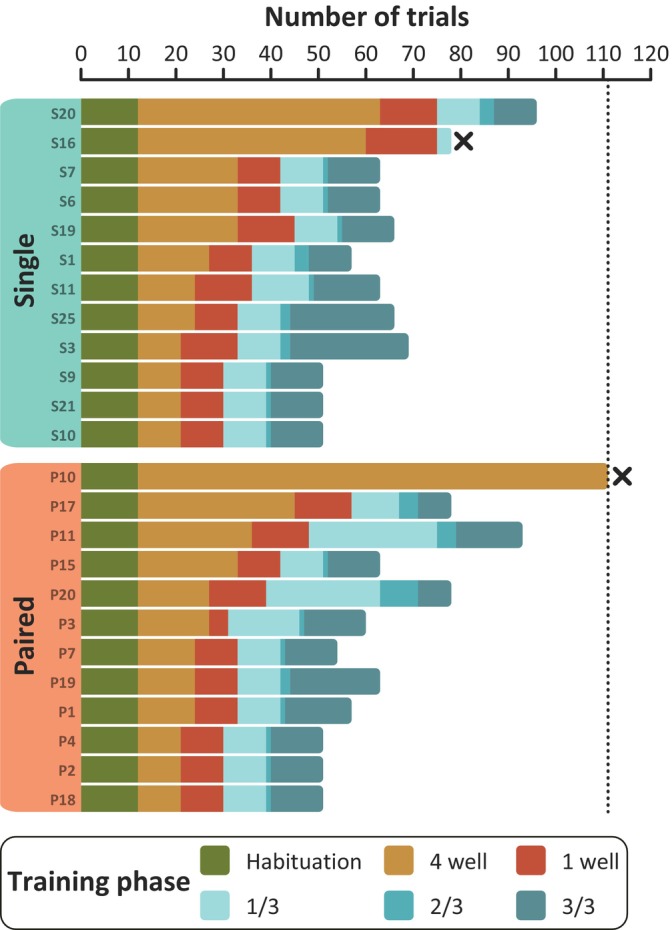
An overview of the number of trials fish needed to complete each of the training phases. Each bar represents one fish, where fish excluded from the final analyses are marked with an “X”. The horizontal dotted line at 111 trials indicates the maximum amount of time fish could spend in the training period before being pushed into the spatial learning task.

Fish were allowed to learn at their own pace throughout the shaping phases, up until a maximum of ~3 weeks (111 trials, fish P10). The only exception to this rule was fish P3 who received more 4 well and 1/3 phases due to not eating a lot of food during her trials, although she finished the shaping otherwise on her own prior to the 3 week cut‐off (Figure 3). Fish P10 and S16 were unable to complete the shaping phases on their own and were pushed directly from the 4 well and 1/3 phases, respectively, into the spatial learning. However, these fish were ultimately excluded from the final analyses presented in this paper. For analyses including these fish, refer to Appendix S2.

#### 2.4.3 Spatial learning

In the spatial learning task, fish had to learn to localize the *Artemia* when presented with two identical green disks in different locations. Both disks concealed a food reward to prevent the fish from relying on olfactory cues to find the *Artemia* (Hara, 1975). For the spatial learning assessment, the left disk (which was used during the shaping) remained the rewarded disk where fish could move the disk to uncover the food reward (three to four *Artemia* per trial). The newly added right disk, however, was the non‐rewarded disk since the disk was fixed in place with a foam plug and could not be moved by the fish. Just as in the previous trials, each trial began by opening the opaque door for ~10 s to allow the fish to observe the training arena, now containing the new non‐rewarded disk. Then the transparent door was opened so the fish could enter the training arena. Fish were given 5 min to solve the task and were scored based on the success of their first disk push. A first push on the rewarded disk was scored as correct (1) and a first push on the non‐rewarded disk was scored as incorrect (0). If fish did not push either disk within the first 5 min of the trial it was scored as non‐choice (NC). For incorrect and non‐choice trials the fish were left to solve the task for an additional 10 min, as described in the training phases. All fish were assessed for a total of 36 trials across six consecutive days, regardless of their success in each trial. As soon as fish completed these 36 trials they were moved into the task reversal in the subsequent training period.

#### 2.4.4 Task reversal

The task reversal phase of the behavioral testing was identical in protocol to the spatial learning task except that the locations of the rewarded and non‐rewarded disks were swapped. The rewarded disk was now located on the right side of the wells‐plate (from the perspective of the experimenter) and the left disk was fixed in place as the non‐rewarded disk. These trials were conducted and scored the same as the spatial learning phase, and again fish performed 36 trials over 6 consecutive days.

After their last task reversal trial, fish were removed from their behavioral tank, euthanized with an overdose of 2‐phenoxyethanol (≥2 mL/L), and rinsed in 1 L of clean system water. Fish were then weighed to determine their wet mass and a Mitutoyo Absolute Digimatic electronic caliber (CD‐15CP) was used to measure their total length, standard length, width (at widest point), and girth (at widest point); these morphological measurements were taken to determine whether housing with a male had any impact on female growth.

### 2.5 Data analysis

Data were collected from 24 fish in total: 12 single and 12 paired fish. However, only 22 (11 single and 11 paired) fish were used for the final analyses due to the inability of two fish to pass the shaping learning criteria. All data analyses were performed in R version 4.3.2 (R Core Team, 2022) in RStudio (Posit team, 2022). All descriptive data (weight, length, number of trials of training needed) were tested for normality using a Shapiro–Wilks normality test. Data which fit the assumption of Gaussian normality were analyzed using a two‐sided *t*‐test, while non‐normal data were analyzed with a Wilcoxon rank sum test.

All behavioral and birth data were analyzed using the *lme4* package (Bates et al., 2015) and model predictions were plot using the *effects* package (Fox, 2003; Fox & Weisberg, 2019). The number of offspring born per brood for both single and paired fish was analyzed using a generalized linear mixed model (GLMM, Poisson) with brood number, treatment group (single or paired), and brood number × treatment group as predictor variables and fish identity as a random variable. For both the spatial and reversal learning tasks, we compared the performance of single and paired fish in:

1. success of the first disk push (1 = success, 0 = fail), where non‐choice trials were treated as incorrect, using a GLMM (binomial) with treatment group (single or paired), trial number in phase, and treatment group × trial number in phase as predictor variables and fish identity as a random variable
2. whether fish make a choice (1 = yes, 0 = no) in a given trial using a GLMM (binomial) with treatment group, trial number in phase, and treatment group × trial number in phase as predictor variables and fish identity as a random variable
3. whether fish who make a choice are successful in their first disk push (1 = success, 0 = fail), using a GLMM (binomial) with treatment group, trial number in phase, and treatment group × trial number in phase as predictor variables and fish identity as a random variable.

All data visualization was done using the *ggplot2* package in Rstudio (Wickham, 2016) and Adobe Illustrator (CC 2023 version 27.2). 

## 3 RESULTS

### 3.1 Offspring per brood is similar for single and paired fish

**FIGURE 4:**
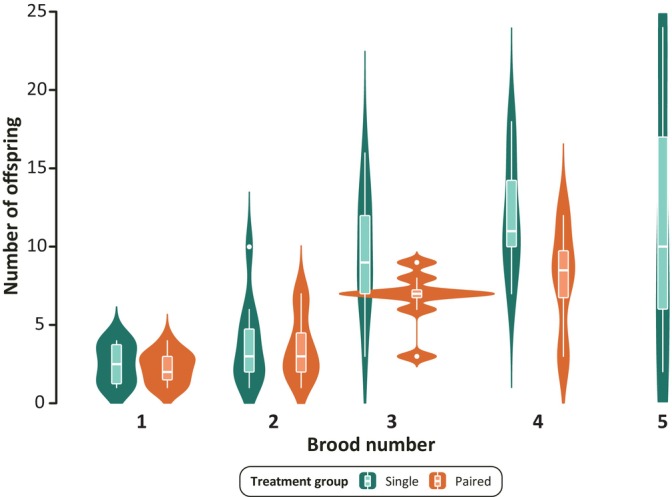
The number of offspring per brood for both single and paired fish (teal and orange, respectively). Box plots indicate the median and interquartile ranges for both groups at each brood number. Underlain violin plots represent the distribution of fish who gave birth to offspring at each brood number.

All paired fish and 11/12 single fish were pregnant during the experimental period. Figure 4 shows the number of offspring per brood (opb) for both single and paired fish over the course of the experiment. As expected, both single and paired fish significantly increased the number of offspring they produced with each successive brood (Table 1) due to the high‐food diet the fish received while housed in the home and experimental tanks. While our model predicts the effect for both groups up to five broods —, as this was the maximum number of broods produced by any fish —, paired fish only produced a maximum of four broods during the experimental period. Paired fish produced an average 4.4 (SD = 2.84) opb while single fish produced 6.79 (SD = 5.51) opb. There was no statistically significant difference in the number of opb between single and paired fish despite their differential access to fresh sperm. Furthermore, paired fish were significantly smaller in weight and standard length, but they did not significantly differ from single fish in width or girth, the two metrics most likely to change during pregnancy (Figure S2).

**TABLE 1:** Outcomes from the statistical model for the number of offspring per brood.

	Estimate	SE	*z* value	*p* value
Number of offspring per brood				
($n_{\text{fish}}=21$, $n_{\mathrm{obs}}=73$)				
Intercept	0.66356	0.19451	3.412	0.000646***
Brood number	0.42104	0.05276	7.981	1.45e−15***
Treatment group (paired)	−0.30900	0.30988	−0.997	0.318688
Brood × treatment group (paired)	0.03964	0.09923	0.399	0.689556

Abbreviations: $n_{\text{fish}}$, the number of fish analyzed; $n_{\mathrm{obs}}$,the number of observations; SE, standard error.

*** *p* < 0.001.

### 3.2 Shaping is similar in both single and paired fish

**FIGURE 5:**
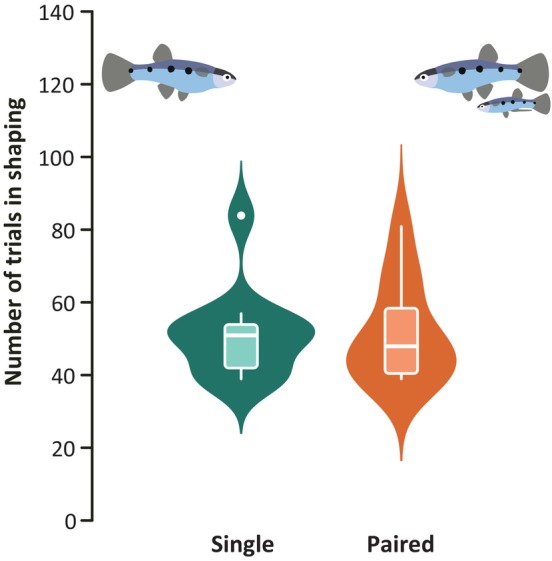
Number of trials required for fish to complete all of the shaping phases. Box plots indicate the median (single, 51 trials; paired, 48 trials) and the interquartile range (single, 12; paired, 18). Underlain violin plots represent the distribution of the fish, where *n* = 11 for both groups.

In the shaping phases, the majority of fish from both groups (11/12 fish per group) met all of the required criteria to pass into the spatial learning task on their own. The two fish unable to pass the learning criteria (S16 and P10) were excluded. Our final analyses and analyses including these fish can be found in Appendix S2. When comparing the total number of trials fish needed to complete all of the shaping phases, there was no significant difference between single and paired fish (Wilcoxon ranked sum test, *n* = 22, *W* = 65, *p* value = 0.7894; Figure 5). In addition, Figure 3 shows there was no substantial difference between the groups in the number of trials fish needed to pass each of the individual subphases.

### 3.3 Single and paired fish show different spatial learning dynamics

**FIGURE 6:**
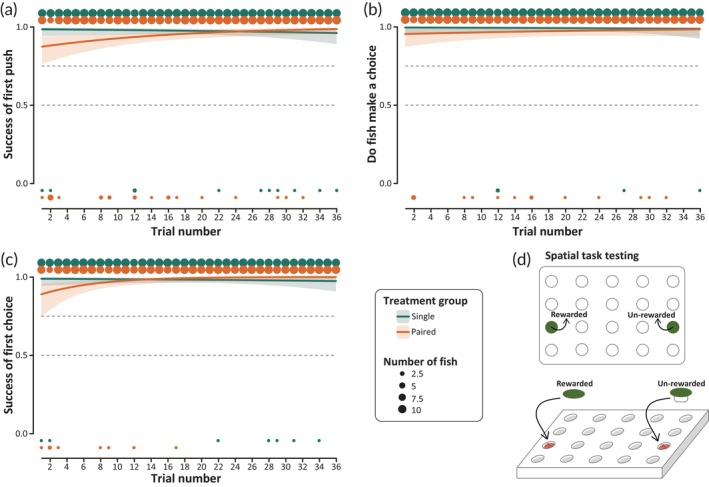
Spatial learning curves as generated by our generalized linear mixed model. Lines indicate the model fit of the effect of the interaction variable (treatment group [single] × trial number), with the lower and upper bounds of the interquartile range. Circles above and below the axes indicate the number of fish from each group who succeeded (1) or failed (0) in each trial, where the area of the circle increases in proportion to the number of fish from 0 to 11. The horizontal dotted lines at 0.5 and 0.75 indicate when fish cross the 50% and 75% learning thresholds, respectively. (a) The success of the first disk push where non‐choice trials are treated as failures. (b) Whether or not fish make a choice in each trial where any disk push is a success and non‐choice is a fail. (c) The success of the first disk push where all non‐choice trials are excluded. (d) A schematic of what the wells‐plate looks like during the spatial learning task.

Both single and paired fish showed a high success rate at the start of the spatial learning task (>75% success). Figure 6a shows the learning curves for both single and paired fish over the course of the 36 spatial learning trials, as generated by our GLMM. Both groups changed their success rate over the course of the trials but differed significantly in terms of how that success changed (Table 2a). Single fish began the spatial learning task with very high success and very low variability between individuals (0.98 ± 0.012 standard error [SE]). However, by trial 29 the success drops slightly (0.97 ± 0.013 [SE]) due to some failed trials performed by four fish (Figure S3a). Paired fish, conversely, began the spatial learning task with a slightly lower success rate and higher variability between individuals (0.87 ± 0.043 [SE]) but were able to improve their performance over the course of the 36 trials, finishing with a success rate slightly above the single fish (0.99 ± 0.008). While this learning difference is significant in our model, we note that the success of both groups was above our 75% learning threshold throughout the entire phase, meaning that both groups were highly successful in the spatial learning task. The variation predicted by our model can therefore be attributed to failed/non‐choice trials performed by only ~4/11 fish per group (Figure S3a), indicating that the practical difference between the groups is minimal.

**TABLE 2:** Outcomes from the statistical models for the spatial learning task.

	Estimate	SE	*z* value	*p* value
(a) Success of the first disk push				
($n_{\text{fish}}=22$, $n_{\mathrm{obs}}=792$)				
Intercept	1.86444	0.40467	4.607	4.08e‐06***
Treatment group (single)	2.29002	0.81732	2.802	0.00508**
Trial number	0.06696	0.02396	2.794	0.00521**
Treatment group (single) × trial number	−0.09387	0.03844	−2.442	0.01461*
(b) Do fish make a choice?				
($n_{\text{fish}}=22$, $n_{\mathrm{obs}}=792$)				
Intercept	3.00280	0.58528	5.131	2.89e‐07***
Treatment group (single)	2.40654	1.33360	1.805	0.0711⋅
Trial number	0.03057	0.02718	1.125	0.2606
Treatment group (single)× trial number	−0.06172	0.05687	−1.085	0.2778
(c) Are fish who make a choice successful?				
($n_{\text{fish}}=22$, $n_{\mathrm{obs}}=774$)				
Intercept	1.91971	0.56021	3.427	0.000611***
Treatment group (single)	2.56525	1.03656	2.475	0.013332*
Trial number	0.16482	0.06128	2.690	0.007153**
Treatment group (single)× trial number	−0.18904	0.07180	−2.633	0.008470**

Abbreviations: $n_{\text{fish}}$, the number of fish analyzed; $n_{\mathrm{obs}}$,the number of observations; SE, standard error.

*** *p* < 0.001.

** *p* < 0.01.

* *p* < 0.05.

To determine whether the differences between single and paired fish observed in the first disk push might be a result of whether or not fish make a choice at all, we analyzed the trials regardless of success (1 = a push on any disk, 0 = non‐choice trial). Figure 6b shows whether or not fish make any choice over the course of the 36 trials. Both single and paired fish consistently make a choice in each of the spatial learning trials, showing high motivation to interact with the wells‐plate, with no statistically significant difference between the groups in whether or not they make a choice (Table 2b). While not significant, there is an ~5‐fold difference in the SE of the effect between the groups at trial 1 (single = 0.0054, paired = 0.0248), with the paired group having a consistently higher SE over the 36 trials. This higher variability between individuals in the paired group is a direct result of the 14 non‐choice trials performed by paired fish over the course of the trials, compared to the four non‐choice trials performed by single fish (Figure S3a).

To remove the impact that non‐choice trials may have on our learning curves in our final models, we analyzed only trials in which fish made a choice. Figure 6c shows those learning curves for single and paired fish, as predicted by our GLMM. Again, fish from both groups exhibited high success over the course of the 36 trials, with both groups performing above the 75% learning threshold. Compared to the previous analyses with the non‐choice trials, the lower and upper limits of the line predicting the performance of paired fish and overall variability in success decreased. However, the difference between single and paired fish remains significant, with single fish performing better at the beginning of the phase and declining in performance in the last couple of trials, while some paired fish were unsuccessful at the beginning of the phase but managed to be successful after only a couple of trials (Table 2c).

### 3.4 Reversal learning performance is similar in both single and paired fish

**FIGURE 7:**
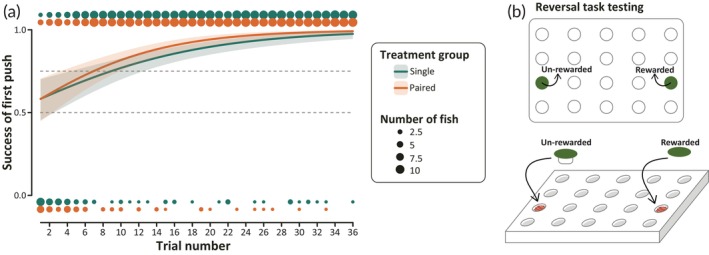
Reversal learning curves as generated by our generalized linear mixed model. Lines indicate the model fit of the effect of the interaction variable (treatment group [single] × trial number), with the lower and upper bounds of the interquartile range. Circles above and below the axes indicate the number of fish from each group who succeeded (1) or failed (0) in each trial, where the area of the circle increases in proportion to the number of fish from 0 to 11. The horizontal dotted lines at 0.5 and 0.75 indicate when fish cross the 50% and 75% learning thresholds, respectively. (a) The success of the first disk push where all non‐choice trials are excluded. (b) A schematic of what the wells‐plate looks like during the reversal learning task, where now the location of the food reward is switched to the left‐finned side of the plate when compared to the spatial learning task.

Both single and paired fish were able to learn the reversal task when the locations of the rewarded and non‐rewarded disks were swapped. Figure 7a shows the success of the first disk push, where all non‐choice trials have been excluded (as described above for the spatial learning task). We also generated a model for the success of the first disk push where non‐choice trials are treated as incorrect (Figure S4a) but there were only 12/792 reversal trials in which fish did not make a choice and there was no significant difference between the groups when comparing whether or not fish make a choice over the 36 reversal trials (Table 3b; Figures S4b and S3b). Unlike in the spatial learning task, there is no statistically significant difference between single and paired fish in performance across the reversal learning task. As expected, fish from both groups show relatively low success rates (0.58 for both single and paired fish) at the start and then improve (0.98 for single and 0.99 for paired at trial 36). Both groups cross the 75% learning threshold after only seven or eight trials, and their success improves significantly over the course of the 36 trials (Table 3c).

**TABLE 3:** Outcomes from the statistical models for the reversal learning task.

	Estimate	SE	*z* value	*p* value
(a) Success of the first disk push				
($n_{\text{fish}}=22$, $n_{\mathrm{obs}}=792$)				
Intercept	0.24593	0.29454	0.835	0.404
Treatment group (single)	−0.09901	0.40295	−0.246	0.806
Trial number	0.11826	0.01988	5.949	2.69e‐09***
Treatment group (single) × trial number	−0.02257	0.02550	−0.885	0.376
(b) Do fish make a choice?				
($n_{\text{fish}}=22$, $n_{\mathrm{obs}}=792$)				
Intercept	5.230977	1.227333	4.262	2.03e‐05***
Treatment group (single)	−1.727045	1.353539	−1.276	0.202
Trial number	0.004749	0.048737	0.097	0.922
Treatment group (single) × trial number	0.067687	0.062091	1.090	0.276
(c) Are fish who make a choice successful?				
($n_{\text{fish}}=22$, $n_{\mathrm{obs}}=780$)				
Intercept	0.20611	0.29216	0.705	0.481
Treatment group (single)	0.03621	0.40166	0.090	0.928
Trial number	0.12969	0.02169	5.979	2.25e‐09***
Treatment group (single) × trial number	−0.03281	0.02749	−1.193	0.233

Abbreviations: $n_{\text{fish}}$, the number of fish analyzed; $n_{\mathrm{obs}}$,the number of observations; SE, standard error.

*** *p* < 0.001.

## 4 DISCUSSION

The aim of this study was to determine if and to what extent coercive mating impacts the cognitive abilities of females. We proposed competing hypotheses on female performance in a spatial learning task: (1) that females from a coercive mating environment would perform better due to their higher propensity to acquire enough food to meet the energy demand of evading males and/or a shift of energy towards better brains to ‘outsmart’ males or (2) that females from a coercive mating environment would perform worse, be it due to elevated levels of stress, negatively impacting brain function, and/or a reallocation of energy towards traits improving evasiveness. However, it seems that coercive mating is neither detrimental nor beneficial for females, as exposure to coercive mating did not show any cognitive impacts on spatial learning and cognitive flexibility.

### 4.1 Coercive mating does not affect female fecundity

Before beginning our behavioral tests, we reared all of our female fish in community tanks with males to ensure that they were pregnant before isolation. During the treatment period and behavioral testing, we tracked each female's reproductive output by counting and collecting their offspring per brood (brood size). Our results show that both single and paired fish significantly increased their brood size over time, as they adjusted to the high‐food environment. This is to be expected since the higher availability of food would enable mothers to allocate increasing resources towards reproduction, allowing for larger brood sizes over time (Reznick et al., 1996). Brood size also continues to increase for both groups during the behavioral testing, indicating there is no detrimental food limitation during this period. In addition, the brood sizes of single versus paired fish did not differ significantly over time in our experiment. In the first of our competing hypotheses on the impacts of coercive mating in *P. gracilis*, we proposed that females would consume more food, either to expend that energy evading males or to reallocate it towards better brains. While we do not directly measure food intake, we aim to utilize female fecundity and morphological measurements to make tentative inferences about female food intake. Our fecundity data shows that either the presence of the male does not impact female food intake or that females prioritize nutrient allocation to offspring production whether or not a male is present. Based on the morphological data presented in Figure S2, we hypothesize the latter to be true, given that while paired fish had significantly lower final weight and standard length when compared to single fish, they did not differ in width and girth. Previous research by Fleuren et al. (2019) found that pregnancy in other superfetatious poeciliids significantly increased the size of the abdomen, specifically the region where we measured width and girth. Our data therefore seem to suggest that paired females do experience a slight energy deficit compared to single females, despite the high‐food diet used in our experiment. This energy deficit may result from the higher energy expenditure paired females have when avoiding male harassment. Since fecundity, width, and girth do not differ between our groups, the energy deficit experienced by the paired females might be reflected in their significantly lower standard length and weight (Figure S2). We therefore propose that in our experiment the slightly food‐limited paired females prioritized resource allocation towards reproduction (i.e. fecundity) rather than towards their own somatic growth (i.e., body weight and standard length).

### 4.2 Minimal impact of coercive mating on spatial learning

Fishes like poeciliids rely on spatial learning and memory to localize both meals and mates in their ever‐changing aquatic ecosystems (Odling‐Smee & Braithwaite, 2003; Salena et al., 2021). It is clear that female poeciliids in coercive mating environments experience a decrease in foraging and feeding efficiency (Griffiths, 1996; Magurran & Seghers, 1994a; Pilastro et al., 2003; Schlupp et al., 2001), which many be attributed to changes in female behavior (i.e., evasive maneuvers, avoidance tactics). Our study therefore aimed to determine if a female's decreased foraging efficiency could also be due in part to changes in cognition in response to coercive mating rather than just changes in behavior. Since spatial learning and memory are particularly important for pregnant individuals, who must find additional nutrients to support their developing offspring, our experiment compares pregnant female performance in a spatial memory task to determine if coercive mating has an impact on female cognition (Reznick et al., 1996).

Our results show an effect of coercive mating on female performance in a spatial memory task (Figure 6c). However, we are very cautious in attributing a biological relevance to this significant interaction effect. Fish success for both groups is above the 75% threshold set by our learning criteria, and observation of the raw data presented in Figure S3a indicates that differences between the two groups can be attributed to failed trials performed by only a handful of individuals. More specifically, 5/11 paired fish performed eight incorrect trials in the first half of the phase and no incorrect trials were performed by any paired fish in the second half of the phase, therefore, over the course of the entire 36 trials, paired fish only selected the incorrect disk about 2.0% of the time. Single fish, however, performed seven incorrect trials (1.8%) over the course of the entire phase, with no consolidation of incorrect trials in the first half of the phase. These failures were performed by more of the single fish, 7/11, although their spread across the entire phase made their effect on the GLMM much weaker. We propose that this differential distribution of incorrect trials ultimately caused the significant difference between our treatment groups. A smaller proportion of paired fish did not remember the location of the correct disk at the beginning of the phase but were able to achieve nearly 100% success by the end of the phase. Meanwhile, a larger proportion of single fish performed incorrect trials, albeit haphazardly throughout the entire phase. Given the >75% success performance in both groups, these data suggest that coercive mating has, at the most, a marginal effect on spatial memory in *P. gracilis*, which does not confer a detrimental effect on paired fish with regards to spatial learning and memory. This implies that the previously reported impacts of coercive mating on female feeding performance can likely be attributed to temporary changes in behavior rather than functional changes in cognition.

We hypothesized that females subject to coercive mating would either perform better to acquire the resources necessary to shift brain function towards “outsmarting” males or perform worse due to stress and the diversion of resources towards evasive maneuvers. While our morphological results indicated that paired females do experience an energy deficit that could be attributed to the presence of coercive males, given that we found no difference in spatial learning performance we cannot attribute this deficit to a reallocation of resources towards the parts of the brain responsible for spatial learning and memory. In addition, any energy which is or has been diverted towards evasiveness does not appear to confer any impacts on female cognition as it pertains to spatial learning. However, these results do not consider energy diversion towards other aspects of the brain, which may be important for females in the presence of coercive males, for example color discrimination and social cognition, as these may relate to mate choice or mating behavior (Bisazza et al., 2001; Cummings, 2015). Future studies may further investigate whether coercive mating directly impacts other forms of cognition to determine how females housed with coercive males may differentially allocate resources compared to females housed as individuals or in female‐only environments. However, from a functional perspective, our data aligns with the fact that female poeciliids will need to find and localize food whether or not males are present, therefore suffering a long‐term detriment to spatial learning as a result of exposure to coercive mating would endanger not only the health of the mother but also the quality and quantity of her offspring.

### 4.3 No impact of coercive mating on cognitive flexibility

Across a wide variety of species, reversal learning has been used to determine an individual's cognitive flexibility by testing their ability to first “un‐learn” a previously rewarded behavior and then acquire or shift their knowledge to learn a newly rewarded behavior (Izquierdo et al., 2017; Tello‐Ramos et al., 2019). Prior research in poeciliids has found that females with larger brains perform better in a reversal learning task than smaller‐brained females, emphasizing the higher complexity of a reversal learning task over a simpler associative task (Buechel et al., 2018). In addition, research in guppies has found some evidence that females are better at reversal learning than males, although the effect size and direction of this sex difference was dependent on the kind of reversal learning task (Lucon‐Xiccato & Bisazza, 2014, 2017; Miletto Petrazzini et al., 2017). In our experiment, we tested female performance in a reversal learning task to determine female cognitive flexibility in spatial learning. In our reversal task, fish were presented with the same green disks as in the previous spatial memory task, except the locations of the rewarded and non‐rewarded disks were swapped. As expected for a reversal learning assay, fish performed poorly at the beginning of the phase as they had to “unlearn” their previously enforced behavior and learn a new task. We found that both single and paired fish improved their reversal learning performance over time, reaching the 75% learning criteria after only ~10 trials. Both groups also showed low variation between individual performances, with variation decreasing over the course of the entire phase. By the end of the phase, fish success in both groups was nearly 100%, with no significant difference in success performance between the groups. Our results indicate that exposure to coercive males does not have an impact on female cognitive flexibility as it pertains to a single instance of reversal learning.

Despite these results, we note that several fish from both our treatment groups (two single and four paired) chose the correct disk in the very first trial of the reversal learning phase, counteracting the previous reinforced paradigm where the fish were taught to always associate the reward with the right‐finned disk (Figure S3b). Given that both single and paired fish exhibited high success performance in the spatial learning task, we cannot assume that these fish simply select the correct disk in the first reversal trial by chance. In our experiments, the rewarded and non‐rewarded disks were visually identical but the non‐rewarded disks were fixed in place due to the addition of a small foam plug glued to the bottom of the disk. We corrected for fish utilizing olfactory cues to locate the food reward by placing food under both the rewarded and non‐rewarded disks, although we acknowledge that there could be other factors which triggered some fish to select the correct disk in the first trial. However, we would like to emphasize that *all* fish from both groups performed at least one incorrect trial during the reversal learning task (Figure S3b). Simply comparing the percentage of incorrect trials performed in the spatial task (1.77% single and 2.02% paired) to those performed in the reversal task (15.15% single and 12.12% paired) we see that fish performed approximately eight times more incorrect trials in the reversal task. These data indicate that even if there were differences in olfactory cues, they likely played no role as all the fish still had to “unlearn” their previously re‐enforced task to learn the new task, exercising their cognitive flexibility in spatial learning. For poeciliid fishes, cognitive flexibility is key to survival in environments where everything, including food availability, turbidity, temperature gradients, water flow, and mate options, can change in relatively short time‐scales (Evans et al., 2011). Food localization, in particular, is essential for pregnant *P. gracilis* living in environments where food availability, as well as location, may change frequently or may be different after having dispersed into a non‐native river system where they must compete with native species (Camacho‐Cervantes et al., 2019; Reznick et al., 1996); mother fish will need to exercise their cognitive flexibility to adjust quickly, securing nutrients for themselves and their offspring.

In the second of our competing hypotheses, we proposed that coercive mating would be detrimental to females since the stress from constant male harassment would have negative impacts on the brain and therefore performance in the spatial and reversal learning tasks. The interaction between chronic stress and cognitive flexibility has been extensively studied in rodent models, where long‐term exposure to stress nearly always impaired an animals' performance in a reversal learning task (as reviewed by Hurtubise and Howland (2017)). In our experiment we saw no effect of coercive mating on female performance in the reversal learning task, indicating that if females experience stress from coercive males this stress may not be detrimental for cognitive flexibility. However, we acknowledge that by analyzing a single instance of reversal learning our experiment may not aptly target cognitive flexibility because it does not account for individual persistence during problem solving and may not represent the sort of cognitive shifts individuals must make in the wild (Audet & Lefebvre, 2017). What is altogether more likely is that female *P. gracilis* are not actively stressed by the presence of coercive males. Instead, females employ some level of adaptive plasticity to cope with male harassment, resulting in a neutral impact of coercive males on female cognition. Therefore, as with spatial learning, evolutionary pressure would favor females with higher cognitive flexibility to allow them to adjust more rapidly to changes in their environments, improving their fitness and indirectly the health of their offspring.

### 4.4 Methodological considerations for social isolation in fish behavioral assays

One of the major hurdles in designing fish behavioral assays is the necessity to test fish as individuals, rather than as a group, when exploring questions outside of schooling or social behaviors. Housing fish individually may result in social isolation, which has been shown to have negative impacts on fish cognition (Brandão et al., 2015; Guo et al., 2022). To avoid socially isolating their fish, many experimenters circumvent this problem by re‐locating fish to the behavioral tanks only during the assay, placing them back into a separate home tank with other individuals in between testing. This is a less than elegant solution as fish will experience additional stress due to handling (Iwama et al., 1998) and will need a period of acclimation before and after every experimental trial, the duration of which may vary between species (Makaras et al., 2021).

In our study, we carefully designed an experimental setup in which we tried to minimize the potentially adverse effects of individual housing as much as possible. First, we housed our fish in a large six‐compartment tank that had no air space between neighboring compartments, allowing the fish to visually interact with their neighboring companions in the adjacent tanks. While social isolation in cichlids has been linked to a decrease in spatial learning performance, the methodology used in that study allowed social fish to view fish in neighboring tanks as a way to remediate social isolation, while isolated fish had no visual neighbors (Brandão et al., 2015). Second, all our tanks were connected to the same filtering system with water continuously being circulated throughout, allowing fish to smell other fish in the system (Solomon, 1977). Third, we housed our fish in the behavioral tanks for the entire duration of the experiment (Buechel et al., 2018; Lucon‐Xiccato & Bisazza, 2014; Vila Pouca et al., 2021; and others), so as to completely avoid handling stress. Fourth, our fish were all reared under the same social conditions and density prior to experimentation to prevent any impacts of early‐life environment on fish cognition (Chapman et al., 2008; Jonsson & Jonsson, 2014). Throughout our experiment, both single and paired fish were housed and tested under low‐density conditions (maximum of one fish per 5 L), which should alleviate any cognitive impacts of the difference in density between the groups (Chapman et al., 2008).

During the treatment period, the single fish in our experiment were housed individually, but had social contact with other fish via the water and by looking through the glass into neighboring tanks. At the start of the behavioral testing, all fish were moved to the behavioral tanks, where they were individually housed for the duration of the experiment, with social contact through the glass in the home compartment (Figure 2d). Given that our experiment found no difference in both task performance and fecundity between single and paired individuals, we conclude that single individuals also did not suffer from being housed individually. This validates the design of our tank set‐up, where fish are housed in conjoining tanks where they have visual contact with neighboring fish and share water from the same recirculating system (i.e., they can see and smell each other), but are unable to directly interact. This set‐up appears to negate or at least counteract some of the impacts of social isolation on associative learning, food localization, and reversal learning (as it relates to a spatial task). Future behavioral research on fishes would benefit from a similar tank set‐up when running experiments with individually housed fish to alleviate stress caused by social isolation or from handling stress during transfer from one tank to another.

## 5 CONCLUSIONS

Our data emphasize female resilience to coercive mating as we did not find any persistent between‐female variations in spatial learning or cognitive flexibility. These findings are particularly relevant for researchers who study poeciliids with coercive mating strategies since this research often necessitates housing males and females together to ensure pregnancy or to study mating behavior. In addition, the aforementioned detrimental effects of coercive mating on foraging and feeding efficiency can be better ascribed to transient changes in female behavior rather than functional changes in cognition. More research is still needed, however, to determine if coercive mating has long‐term impacts on other aspects of female cognition, such as social cognition or color discrimination, which may relate more directly to mate choice or mating behavior. Overall, this study asserts three main conclusions about the impacts of coercive mating on female *Poeciliopsis gracilis* and their cognition: (1) coercive mating does not impact the amount of offspring females can produce as long as food availability is high, (2) spatial learning is marginally impacted in females exposed to coercive mating although success is high enough as to not be detrimental, and (3) exposure to coercive mating had no impact on performance in a reversal learning task, indicating that it does not confer impacts on cognitive flexibility.

## AUTHOR CONTRIBUTIONS

T.R.E. conceived of the study, helped with generating the data, performed data analysis, wrote the manuscript, and prepared it for submission. R.M.H.W.H. did the majority of the data generation and helped with methodological design. J.L.vL. helped conceive the study, contributed to study design and methodology, helped with data acquisition, and provided feedback on drafts of the manuscript. A. Kotrschal, A. Korosi, and B.J.A.P. generated ideas for the study design and research questions, and provided feedback on the manuscript. B.J.A.P. secured the required funding for this project. All the authors read and approved the final manuscript.

## FUNDING INFORMATION

This study was funded by a Vidi grant (864.14.008) from the Netherlands Organization for Scientific Research (NWO) and HORIZON‐RIA grant (HLTH‐2021‐STAYHLTH‐01: 101057390) awarded to B.J.A.P.

## Supporting information


**APPENDIX S1.** Metadata.

**APPENDIX S2.** Code and analyses.

**FIGURE S1.** A schematic of the ranks generated for fish selection, based on the average number of offspring per brood for each fish. The ranks are given by the number listed at the top of each stack. Ranges running along the *y* axis indicate the average number of offspring per brood for fish in those ranks, where rank 1 has a range of 0 to 1 but not including 1, rank 2 has a range of 1 to 2 but not including 2 and so on. Fish were ranked within their assigned treatment group (single, above; paired, below). Fish which were not selected for the experiment are shown in gray and selected fish are shown in teal and orange for single and paired fish, respectively.

**FIGURE S2.** Morphological characteristics of the fish in this study as measured at the end of the experiment. (a) Weight of the fish in grams (*t* = 2.6561, *df* = 18.04, *p* value = 0.01606). (b) Standard length of the fish in millimeters (*W* = 101, *p* value = 0.006634). (c) Width of the fish in millimeters at the widest point (*W* = 88, *p* value = 0.07589). (d) Girth of the fish in millimeters at the widest point (*W* = 87, *p* value = 0.08795).

**FIGURE S3.** Heat maps representing the raw data from the spatial and reversal learning tasks. Fish excluded from the final analyses are marked with an “X" next to their identity code. (a) The success of each fish on their first disk push during the spatial learning task, where all failures (0) are shown in black, non‐choice trials are shown in gray, and successes (1) are shown in teal or orange for single or paired fish, respectively. (b) The success of each fish on their first disk push in the reversal learning task, where all failures (0) are shown in black, non‐choice trials are shown in gray, and successes (1) are shown in teal or orange for single or paired fish, respectively.

**FIGURE S4.** Reversal learning curves as generated by our generalized linear mixed models. Lines indicate the model fit of the effect of the interaction variable (treatment group [single] × trial number) with the lower and upper bounds of the interquartile range. Circles above and below the axes indicate the number of fish from each group who succeed (1) or fail (0) in each trial, where the area of the circle increases in proportion to the number of fish from 0 to 11. (a) The success of the first disk push, where non‐choice trials are treated as failures. (b) Whether or not fish make a choice in each trial, where any disk push is a success and non‐choice is a fail.

## Data Availability

The data, code, and analyses that support the findings of this study are openly available in Figshare at: 10.6084/m9.figshare.23578941 (data) and 10.6084/m9.figshare.23579178 (code & analyses).
