# Supplementary material for: Coercive mating has no impact on spatial learning, cognitive flexibility, and fecundity in female porthole livebearers (Poeciliopsis gracilis)
Source: J Fish Biol. 2024 Feb 25;107(4):1106–21. doi: 10.1111/jfb.15696 (PMC12536062; doi:10.1111/jfb.15696)
Supplement: Supplementary file 1 — APPENDIX S1. Metadata. [file JFB-107-1106-s001.html]

Appendix S1– Metadata: Coercive mating has no impact on spatial learning, cognitive flexibility and fecundity in female porthole livebearers (Poeciliopsis gracilis)


Code 

- Show All Code
- Hide All Code

# **Appendix S1– Metadata:** Coercive mating has no impact on spatial learning, cognitive flexibility and fecundity in female porthole livebearers (*Poeciliopsis gracilis*)

#### Ernst TR, Hogers RMHW, Korosi A, van Leeuwen JL, Kotrschal A, & Pollux BJA

# 1 General Information:

This is the metadata file for all data utilized in the paper
“Coercive mating has no impact on spatial learning, cognitive
flexibility and fecundity in female porthole livebearers
(*Poeciliopsis gracilis*)”. All code and analyses performed on
this data can be found in Appendix S2. The files included
in the data set are as follows:

| Filename | Description |
| --- | --- |
| 01\_fish\_profiles\_MBP2.csv | An overview of all individual fish data, including morphometric data and fish identities. See below for additional metadata. |
| 02\_habituation\_MBP2.csv | Behavioral data collected per fish during the habituation phase of the experiment. See below for additional metadata. |
| 03\_pre\_learning\_MBP2.csv | Behavioral data collected per fish during the pre-learning/association learning phases of the experiment. See below for additional metadata. |
| 04\_spatial\_learning\_MBP2.csv | Behavioral data collected per fish during the spatial learning task. See below for additional metadata. |
| 05\_reversal\_learning\_MBP2.csv | Behavioral data collected per fish during the reversal learning task. See below for additional metadata. |
| 06\_birth\_data\_MBP2.csv | An overview of the number of offspring per brood produced by each fish in the experiment. See below for additional metadata. |
| 07\_trialsprelearning\_MBP2.csv | An overview of the number of trials fish needed to pass each of the pre-learning/associative learning phases in the experiment. See below for additional metadata. |

The following sections contain the individual metadata for each file,
describing what kind of data is indicated by each column name.

**NOTE:** All data files include fish identity codes
where V# indicates a single fish and P# indicates a paired fish. These
labels were changed in the final publication for reader-clarity but
remain here since they were used to run all of the analyses.

# 2 Fish Profiles

Metadata for the data file: 01\_fish\_profiles\_MBP2.csv.

| Column Name | Description |
| --- | --- |
| fish\_code | fish identity code |
| status | experimental group of the fish - paired OR single |
| system | tank system of the fish - left OR right |
| batch | batch # determined on when fish started the experiment |
| trials\_prelearning | # trials fish needed to complete pre-learning |
| start\_fw\_trainingmoment | training moment of the first forward task |
| start\_rv\_trainingmoment | training moment of the first reverse task |
| end\_trainingmoment | training moment of when the fish finished the entire experiment |
| weight | wet weight of the fish in grams |
| total\_length | total length of the fish in mm - at end of the experiment |
| standard\_length | standard length of the fish in mm - at end of the experiment |
| width | width of the fish in mm - at end of the experiment |
| girth | girth of the fish in mm - at end of the experiment |
| ovary\_status | whether there were visible embryos present in the ovary at the time of dissection (end of the experiment) |
| repro\_status\_task\_testing | whether fish gave birth during the forward task |
| repro\_status\_reversal | whether fish gave birth during the reversal task |
| repro\_status\_alltesting | whether fish gave birth at any point during the experiment (including habituation, pre-learning, forward & reversal) |

# 3 Habituation

Metadata for the data file: 02\_habituation\_MBP2.csv.

| Column Name | Description |
| --- | --- |
| fish\_code | fish identity code |
| phase | phase of the experiment |
| training\_moment | the time of day in which the trial took place; morning OR afternoon |
| trial\_inphase | the trial number within this phase of the experiment |
| start | time when the fish started the trial |
| finish | time when the fish finished the trial, where NA indicates that the food was left overnight |
| trial\_duration | number of hours the fish had to eat from the plate during the trial |
| 2hr\_check | whether fish had eaten after 2hr or overnight; 1 = yes, 0 = no |
| notes | any notes about the fish during the trial |

# 4 Pre-learning & Associative Learning

## 4.1 Pre-learning performance

Metadata for the data file: 03\_pre\_learning\_MBP2.csv.

| Column Name | Description |
| --- | --- |
| fish\_code | fish identity code |
| phase | phase of the trial |
| subphase | subphase of the trial: 4well, 1well, 1/3, 2/3, or 3/3 |
| training\_moment | time of day in which the trial took place: morning OR afternoon |
| trial\_inphase | trial number within this phase |
| 5min | whether fish ate the food within the first 5min: 1 = yes, 0 = no |
| 10min | whether fish ate the food in the subsequent 10min: 1 = yes, 0 = no |
| 15min | whether fish ate the food within the total 15min of the trial: 1 = yes, 0 = no |
| food\_eaten | the amount of food eaten by the fish: 0 = none, 1 = small amount, 2 = medium amount, 3 = completely eaten |
| births | did fish give birth directly before this trial (ie. Were babies found in the tank): 1 = yes, 0 = no |
| help | did fish receive help to complete the trial: 1 = yes, 0 = no |
| notes | any notes about the fish during this trial |

## 4.2 Pre-learning trial overview

Metadata for the data file: 07\_trialsprelearning\_MBP2.csv.

| Column Name | Description |
| --- | --- |
| fish\_code | fish identity |
| phase\_learning | phase of the experiment |
| trials | number of trials performed by the fish in the corresponding phase |
| trials\_prelearning | total number of trials of pre-learning performed by the fish |

# 5 Spatial & Reversal Learning

Metadata for the data files: 04\_spatial\_learning\_MBP2.csv and
05\_reversal\_learning\_MBP2.csv.

| Column Name | Description |
| --- | --- |
| fish\_code | fish identity code |
| phase | phase of the trial |
| training\_moment | time of day in which the trial took place: morning OR afternoon |
| trial\_inphase | trial number within this phase |
| disk\_push | whether the first push of the fish was correct: -1 = incorrect, 0 = no choice, 1 = correct |
| success\_push | whether the first push of the fish was correct (where no choice is treated as incorrect): 0 = incorrect or no choice, 1 = correct |
| success\_choice\_push | whether the first push of the fish was correct (where no choice is omitted by inserting NA): 0 = incorrect, 1 = correct |
| choice\_push | whether the fish pushed any disk during the trial (regardless of whether it is the correct disk): 1 = yes, 0 = no |
| time\_push | the time in minutes during the trial at which the fish made their first push (regardless of whether it is the correct disk): value is NA if fish never pushed a disk |
| 5min | whether fish ate the food within the first 5min: 1 = yes, 0 = no |
| 10min | whether fish ate the food in the subsequent 10min: 1 = yes, 0 = no |
| 15min | whether fish ate the food within the total 15min of the trial: 1 = yes, 0 = no |
| food\_eaten | the amount of food eaten by the fish: 0 = none, 1 = small amount, 2 = medium amount, 3 = completely eaten |
| births | did fish give birth directly before this trial (ie. were babies found in the tank): 1 = yes, 0 = no |
| help | did fish receive help to complete the trial: 1 = yes, 0 = no |
| notes | any notes about the fish during this trial |

# 6 Birth Data

Metadata for the data file: 06\_birth\_data\_MBP2.csv.

| Column Name | Description |
| --- | --- |
| fish\_code | fish identity code |
| brood | the brood number for that fish (ie. 1st, 2nd, 3rd brood etc.) |
| babies | the number of babies born in that brood |
| IBI | the length of time in days between this brood and the last brood |
