# Supplementary material for: Coercive mating has no impact on spatial learning, cognitive flexibility, and fecundity in female porthole livebearers (Poeciliopsis gracilis)
Source: J Fish Biol. 2024 Feb 25;107(4):1106–21. doi: 10.1111/jfb.15696 (PMC12536062; doi:10.1111/jfb.15696)
Supplement: Supplementary file 2 — APPENDIX S2. Code and analyses. [file JFB-107-1106-s005.html]

Appendix S2– Code & Analyses: Coercive mating has no impact on spatial learning, cognitive flexibility and fecundity in female porthole livebearers (Poeciliopsis gracilis)


Code 

- Show All Code
- Hide All Code

# **Appendix S2– Code & Analyses:** Coercive mating has no impact on spatial learning, cognitive flexibility and fecundity in female porthole livebearers (*Poeciliopsis gracilis*)

#### Ernst TR, Hogers RMHW, Korosi A, van Leeuwen JL, Kotrschal A, & Pollux BJA

```
knitr::opts_chunk$set(echo = TRUE)

## CLEAR WORKSPACE ----
rm(list=ls())

## LOAD PACKAGES ----
library(shiny)
library(lme4)
library(effects)
library(ggplot2)
library(cowplot)
library(plyr)
library(dplyr)
library(boot)
library(tidyr)
library(Rmisc)
library(RColorBrewer)
library(kableExtra)
library(ggstance)
library(ggpubr)
library(ggsignif)
library(ggh4x)

## READ DATA FILES ----
fish_profiles <- read.csv("01_fish_profiles_MBP2.csv", sep=";", dec = ",")
birth_data <- read.csv("06_birth_data_MBP2.csv", sep=";", dec = ",")
trials_learning <- read.csv("07_trialsprelearning_MBP2.csv", sep=";", dec = ",")
task_testing <- read.csv("04_spatial_learning_MBP2.csv", sep=";", dec = ",")
reversal_learning <- read.csv("05_reversal_learning_MBP2.csv", sep=";", dec = ",")

## COMBINE RELEVANT DATA FILES ---
fish_birth_data_temp <- birth_data %>% inner_join(fish_profiles, by = "fish_code")
fish_birth_data_FINAL <- fish_birth_data_temp %>% select(fish_code, brood:batch, weight:ovary_status)

trials_pre_learning_fish <- trials_learning %>% inner_join(fish_profiles, by = "fish_code")
trials_pre_learning_FINAL <- trials_pre_learning_fish %>% select(fish_code:trials, status)

task_testing_repro <- task_testing %>% inner_join(fish_profiles, by = "fish_code")
task_testing_FINAL <- task_testing_repro %>% select(fish_code, status, training_moment:choice_push, time_push_min:X15min)

reversal_learning_repro <- reversal_learning %>% inner_join(fish_profiles, by = "fish_code")
reversal_learning_FINAL <- reversal_learning_repro %>% select(fish_code, status, training_moment:choice_push, time_push_min:X15min)

## REORDER LEVELS FOR RELEVANT DATA FILES ---
fish_birth_data_FINAL$status <- factor(fish_birth_data_FINAL$status, levels = c("single", "paired"))
fish_profiles$status <- factor(fish_profiles$status, levels = c("single", "paired"))
trials_pre_learning_FINAL$status <- factor(trials_pre_learning_FINAL$status, levels = c("single", "paired"))
```

# 1 General Information

This file contains all the code and analysis for the data utilized in
the paper “Coercive mating has no impact on spatial learning, cognitive
flexibility and fecundity in female porthole livebearers
(*Poeciliopsis gracilis*)”. All data files (including metadata)
can be found in Appendix
S1.

**Note on excluding non-performing fish:** Fish P10 and
S16 were excluded from the final analyses because they did not reach the
established learning criteria prior to the start of spatial learning.
The analyses shown here exclude these fish by default as this is what is
reported in the final paper. If you would like to run the code
*including* these individuals simply exclude the following code
chunk.

```
#adjusts the analyzed dataframes to exclude P10 and S16 (non-performing fish) when necessary:
fish_birth_data_FINAL <- subset(fish_birth_data_FINAL, fish_code != "P10" & fish_code != "V16")
fish_profiles_FINAL <- subset(fish_profiles, fish_code != "P10" & fish_code != "V16")
task_testing_FINAL <- subset(task_testing_FINAL, fish_code != "P10" & fish_code != "V16")
reversal_learning_FINAL <- subset(reversal_learning_FINAL, fish_code != "P10" & fish_code != "V16")
```

# 2 Fecundity

Determining whether coercive mating (single or paired) had an impact
on the number of offspring fish produced with each successive brood.

## 2.1 GLM

```
#response variable: number of offspring (poisson; # of babies cannot be negative)
#potential predictors: brood, status (single or paired), the interaction between brood and status, and fish identity as a random variable.
baby_number_glm1 <- glmer(babies ~ brood + status + brood:status + (1|fish_code),
                             family= poisson, data = fish_birth_data_FINAL)
summary(baby_number_glm1)
```

```
## Generalized linear mixed model fit by maximum likelihood (Laplace
##   Approximation) [glmerMod]
##  Family: poisson  ( log )
## Formula: babies ~ brood + status + brood:status + (1 | fish_code)
##    Data: fish_birth_data_FINAL
## 
##      AIC      BIC   logLik deviance df.resid 
##    348.5    360.0   -169.2    338.5       68 
## 
## Scaled residuals: 
##      Min       1Q   Median       3Q      Max 
## -2.75671 -0.68296  0.04586  0.59561  2.34847 
## 
## Random effects:
##  Groups    Name        Variance Std.Dev.
##  fish_code (Intercept) 0.07371  0.2715  
## Number of obs: 73, groups:  fish_code, 21
## 
## Fixed effects:
##                    Estimate Std. Error z value Pr(>|z|)    
## (Intercept)         0.66356    0.19451   3.412 0.000646 ***
## brood               0.42104    0.05276   7.981 1.45e-15 ***
## statuspaired       -0.30900    0.30988  -0.997 0.318688    
## brood:statuspaired  0.03964    0.09923   0.399 0.689556    
## ---
## Signif. codes:  0 '***' 0.001 '**' 0.01 '*' 0.05 '.' 0.1 ' ' 1
## 
## Correlation of Fixed Effects:
##             (Intr) brood  sttspr
## brood       -0.821              
## statuspaird -0.620  0.515       
## brd:sttsprd  0.433 -0.531 -0.850
```

## 2.2 Plot

The final plot was brought into Adobe Illustrator to adjust the
overlapping of the violin plots and to fix the offsetting of the boxplot
and the violin plot for the final brood number.

```
#Determine the marginal effect of brood:status on offspring number (babies) according to our model (where NA values are NOT removed) and convert this to a data frame.
baby_number_glm1_fit <- effect('brood:status', baby_number_glm1, xlevels = 6, na.rm=FALSE) %>% as.data.frame()

#reorder the levels of single & paired in our new fit dataframe.
baby_number_glm1_fit$status <- factor(baby_number_glm1_fit$status, levels = c("single", "paired"))
#change the class of our status variable
fish_birth_data_FINAL$status <- as.character(as.numeric(fish_birth_data_FINAL$status))

#make the plot
babynumber_plot <- ggplot(fish_birth_data_FINAL, aes(x = brood, y = babies, colour = status)) +
  geom_violin(data = fish_birth_data_FINAL, aes(factor(brood), babies, colour = status, fill= factor(status)), position=position_dodge(width=0.9), width = 4, scale = "area", trim = FALSE)  + #makes the violin plots
  geom_boxplot(data = fish_birth_data_FINAL, aes(factor(brood), babies, fill= factor(status)), position=position_dodge(width=0.9, preserve = "single"), width = 0.25, colour = "white", outlier.color="white") + #overlays the boxplots over the violin plots
  scale_y_continuous(breaks = seq(0, 25, 5), limits = c(0,25), expand = c(0, 0)) + #sets the scaling parameters for the y axis
  scale_x_discrete(limits = c("1","2", "3", "4", "5"), expand = expansion(add = c(0,0))) + #sets the scaling parameters for the x axis
  geom_segment(aes(x = 0, y = 0, xend = 0, yend = 25), lineend = "round", colour = "#1E1F17") + #redraws the y-axis
  labs(title = "number of offspring per brood", x = "brood number", y="number of offspring") + #defines the x and y axis labels
  scale_fill_manual(name = "status", values=c("#227266", "#D86631")) + #defines the fill aesthetics in the legend
  scale_colour_manual(name = "status", values=c("#227266", "#D86631")) + #defines the color aesthetics in the legend
  theme(plot.title = element_text(size = 12, hjust = 0.5),
        text = element_text(size = 16, colour = "#1E1F17", family = "sans"),
        axis.title.x = element_text(),
        axis.title.y = element_text(vjust = 1.5),
        axis.line = element_blank(),
        axis.ticks.length = unit(2, "mm"),
        axis.ticks.x = element_blank(),
        axis.ticks = element_line(colour = "#1E1F17",lineend = "round"),
        panel.background = element_blank(),
        legend.key = element_blank(),
        legend.position = "none") #controls theme elements for the entire plot
babynumber_plot
```

# 3 Morphometrics

Each morphometric plot was generated individually and then arranged
into a grid in Adobe Illustrator.

## 3.1 Weight

Determining whether coercive mating (single or paired) had an impact
on the weight of the fish at the end of the experiment. Normality of the
data is first assessed using a Shapiro Wilk’s test and visualized with a
density plot. Summary statistics, t-test results and final plots are
shown below.

```
#test to see if the data are normally distributed:
#If p>0.05 then data ARE normal. If p<0.05 then data are NOT normal.
#p>0.05 so data ARE normal!
shapiro.test(fish_profiles$weight)
```

```
## 
##  Shapiro-Wilk normality test
## 
## data:  fish_profiles$weight
## W = 0.96267, p-value = 0.4943
```

```
ggdensity(fish_profiles$weight)
```

```
#compute summary statistics per group (single vs paired):
group_by(fish_profiles, status) %>% summarise(count = n(), 
                                              median = median(weight, na.rm = TRUE), 
                                              IQR = IQR(weight, na.rm = TRUE))
```

```
## # A tibble: 3 × 4
##   status count median    IQR
##   <fct>  <int>  <dbl>  <dbl>
## 1 single    12   1.89  0.320
## 2 paired    12   1.31  0.46 
## 3 <NA>     288  NA    NA
```

```
#two-sided t-test:
t.test(weight ~ status, data = fish_profiles, alternative = "two.sided")
```

```
## 
##  Welch Two Sample t-test
## 
## data:  weight by status
## t = 2.9697, df = 20.524, p-value = 0.007432
## alternative hypothesis: true difference in means between group single and group paired is not equal to 0
## 95 percent confidence interval:
##  0.1427481 0.8129186
## sample estimates:
## mean in group single mean in group paired 
##             1.821417             1.343583
```

```
#p<0.05 there IS a significant difference between the two groups
```

The final plot was taken into Adobe Illustrator to add significance
bars, remove the NA column, and make aesthetic changes.

```
#plot the composite graph:
weight_plot <- ggplot() +
  geom_violin(data = fish_profiles, aes(status, weight, colour = status, fill = status), trim = FALSE)  + #makes the violin plots
  geom_boxplot(data = fish_profiles, aes(status, weight, fill= status), width=0.2, colour = "white", outlier.color="white") + #overlays the boxplots over the violin plots
  scale_y_continuous(breaks = seq(0, 3, 0.5), limits = c(0,3), expand = c(0, 0)) + #sets the scaling parameters for the y axis
  scale_x_discrete(expand = expansion(add = c(0,1))) + #sets the scaling parameters for the x axis
  geom_segment(aes(x = 0, y = 0, xend = 0, yend = 3), lineend = "round", colour = "#1E1F17") + #redraws the y-axis
  labs(title = "weight of the fish", y="weight (g)") + #defines the x and y axis labels
  scale_fill_manual(name = "status", values=c("#227266", "#D86631")) + #defines the fill aesthetics in the legend
  scale_colour_manual(name = "status", values=c("#227266", "#D86631")) + #defines the color aesthetics in the legend
  theme(plot.title = element_text(size = 12, hjust = 0.5),
        text = element_text(size = 16, colour = "#1E1F17", family = "sans"),
        axis.title.x = element_blank(),
        axis.title.y = element_text(vjust = 1.5),
        axis.line = element_blank(),
        axis.ticks.length = unit(2, "mm"),
        axis.ticks.x = element_blank(),
        axis.ticks = element_line(colour = "#1E1F17",lineend = "round"),
        panel.background = element_blank(),
        legend.key = element_blank(),
        legend.position = "none") #controls theme elements for the entire plot
weight_plot
```

## 3.2 Length

Determining whether coercive mating (single or paired) had an impact
on the length of the fish at the end of the experiment. Normality of the
data is first assessed using a Shapiro Wilk’s test and visualized with a
density plot. Summary statistics, wilcox test results and final plots
are shown below.

```
#test to see if the data are normally distributed:
#If p>0.05 then data ARE normal. If p<0.05 then data are NOT normal.
#p>0.05 so data ARE normal!
shapiro.test(fish_profiles$standard_length)
```

```
## 
##  Shapiro-Wilk normality test
## 
## data:  fish_profiles$standard_length
## W = 0.91579, p-value = 0.04717
```

```
ggdensity(fish_profiles$standard_length)
```

```
#compute summary statistics per group (single vs paired):
group_by(fish_profiles, status) %>% summarise(count = n(), 
                                              median = median(standard_length, na.rm = TRUE), 
                                              IQR = IQR(standard_length, na.rm = TRUE))
```

```
## # A tibble: 3 × 4
##   status count median   IQR
##   <fct>  <int>  <dbl> <dbl>
## 1 single    12   43.4 11.1 
## 2 paired    12   37.0  4.42
## 3 <NA>     288   NA   NA
```

```
#two-sided t-test:
wilcox.test(standard_length ~ status, data = fish_profiles, alternative = "two.sided")
```

```
## 
##  Wilcoxon rank sum exact test
## 
## data:  standard_length by status
## W = 121, p-value = 0.003637
## alternative hypothesis: true location shift is not equal to 0
```

```
#p<0.05 there IS a significant difference between the two groups
```

The final plot was taken into Adobe Illustrator to add significance
bars, remove the NA column, and make aesthetic changes.

```
#plot the composite graph:
standard_length_plot <- ggplot() +
  geom_violin(data = fish_profiles, aes(status, standard_length, colour = status, fill = status), trim = FALSE)  + #makes the violin plots
  geom_boxplot(data = fish_profiles, aes(status, standard_length, fill= status), width=0.2, colour = "white", outlier.color="white") + #overlays the boxplots over the violin plots
  scale_y_continuous(breaks = seq(25, 65, 5), limits = c(25,65), expand = c(0, 0)) + #sets the scaling parameters for the y axis
  scale_x_discrete(expand = expansion(add = c(0,1))) + #sets the scaling parameters for the x axis
  geom_segment(aes(x = 0, y = 25, xend = 0, yend = 65), lineend = "round", colour = "#1E1F17") + #redraws the y-axis
  labs(title = "standard length of the fish", y="standard length (mm)") + #defines the x and y axis labels
  scale_fill_manual(name = "status", values=c("#227266", "#D86631")) + #defines the fill aesthetics in the legend
  scale_colour_manual(name = "status", values=c("#227266", "#D86631")) + #defines the color aesthetics in the legend
  theme(plot.title = element_text(size = 12, hjust = 0.5),
        text = element_text(size = 16, colour = "#1E1F17", family = "sans"),
        axis.title.x = element_blank(),
        axis.title.y = element_text(vjust = 1.5),
        axis.line = element_blank(),
        axis.ticks.length = unit(2, "mm"),
        axis.ticks.x = element_blank(),
        axis.ticks = element_line(colour = "#1E1F17",lineend = "round"),
        panel.background = element_blank(),
        legend.key = element_blank(),
        legend.position = "none") #controls theme elements for the entire plot
standard_length_plot
```

## 3.3 Width

Determining whether coercive mating (single or paired) had an impact
on the width of the fish at the end of the experiment. Normality of the
data is first assessed using a Shapiro Wilk’s test and visualized with a
density plot. Summary statistics, t-test results and final plots are
shown below.

```
#test to see if the data is normally distributed:
shapiro.test(fish_profiles_FINAL$width)
```

```
## 
##  Shapiro-Wilk normality test
## 
## data:  fish_profiles_FINAL$width
## W = 0.90729, p-value = 0.0417
```

```
ggdensity(fish_profiles_FINAL$width)
```

```
#If p>0.05 then data ARE normal. If p<0.05 then data are NOT normal.
#p>0.05 so data ARE NOT normal! (NOTE: this data is normal with the inclusion of S16 & P10)

#compute summary statistics per group (single vs paired):
group_by(fish_profiles_FINAL, status) %>% summarise(count = n(), 
                                              median = median(width, na.rm = TRUE), 
                                              IQR = IQR(width, na.rm = TRUE))
```

```
## # A tibble: 3 × 4
##   status count median   IQR
##   <fct>  <int>  <dbl> <dbl>
## 1 single    11   12.1  1.16
## 2 paired    11   11.0  1.41
## 3 <NA>     288   NA   NA
```

```
#two-sided t-test:
wilcox.test(width ~ status, data = fish_profiles_FINAL, alternative = "two.sided")
```

```
## 
##  Wilcoxon rank sum exact test
## 
## data:  width by status
## W = 88, p-value = 0.07589
## alternative hypothesis: true location shift is not equal to 0
```

```
#p>0.05 there is NOT a significant difference between the two groups
```

The final plot was taken into Adobe Illustrator to add significance
bars, remove the NA column, and make aesthetic changes.

```
#plot the composite graph:
width_plot <- ggplot() +
  geom_violin(data = fish_profiles, aes(status, width, colour = status, fill = status), trim = FALSE)  + #makes the violin plots
  geom_boxplot(data = fish_profiles, aes(status, width, fill= status), width=0.2, colour = "white", outlier.color="white") + #overlays the boxplots over the violin plots
  scale_y_continuous(breaks = seq(5, 25, 5), limits = c(5,25), expand = c(0, 0)) + #sets the scaling parameters for the y axis
  scale_x_discrete(expand = expansion(add = c(0,1))) + #sets the scaling parameters for the x axis
  geom_segment(aes(x = 0, y = 5, xend = 0, yend = 25), lineend = "round", colour = "#1E1F17") + #redraws the y-axis
  labs(title = "width of the fish", y="width (mm)") + #defines the x and y axis labels
  scale_fill_manual(name = "status", values=c("#227266", "#D86631")) + #defines the fill aesthetics in the legend
  scale_colour_manual(name = "status", values=c("#227266", "#D86631")) + #defines the color aesthetics in the legend
  theme(plot.title = element_text(size = 12, hjust = 0.5),
        text = element_text(size = 16, colour = "#1E1F17", family = "sans"),
        axis.title.x = element_blank(),
        axis.title.y = element_text(vjust = 1.5),
        axis.line = element_blank(),
        axis.ticks.length = unit(2, "mm"),
        axis.ticks.x = element_blank(),
        axis.ticks = element_line(colour = "#1E1F17",lineend = "round"),
        panel.background = element_blank(),
        legend.key = element_blank(),
        legend.position = "none") #controls theme elements for the entire plot
width_plot
```

## 3.4 Girth

Determining whether coercive mating (single or paired) had an impact
on the girth of the fish at the end of the experiment. Normality of the
data is first assessed using a Shapiro Wilk’s test and visualized with a
density plot. Summary statistics, t-test results and final plots are
shown below.

```
#test to see if the data is normally distributed:
shapiro.test(fish_profiles_FINAL$girth)
```

```
## 
##  Shapiro-Wilk normality test
## 
## data:  fish_profiles_FINAL$girth
## W = 0.91111, p-value = 0.04988
```

```
ggdensity(fish_profiles_FINAL$girth)
```

```
#If p>0.05 then data ARE normal. If p<0.05 then data are NOT normal.
#p>0.05 so data ARE NOT normal! (NOTE: This data is normal with the inclusion of S16 & P10)

#compute summary statistics per group (single vs paired):
group_by(fish_profiles_FINAL, status) %>% summarise(count = n(), 
                                              median = median(girth, na.rm = TRUE), 
                                              IQR = IQR(girth, na.rm = TRUE))
```

```
## # A tibble: 3 × 4
##   status count median    IQR
##   <fct>  <int>  <dbl>  <dbl>
## 1 single    11   8.92  0.805
## 2 paired    11   8.2   1.07 
## 3 <NA>     288  NA    NA
```

```
#two-sided t-test:
wilcox.test(girth ~ status, data = fish_profiles_FINAL, alternative = "two.sided")
```

```
## 
##  Wilcoxon rank sum exact test
## 
## data:  girth by status
## W = 87, p-value = 0.08795
## alternative hypothesis: true location shift is not equal to 0
```

```
#p>0.05 there is NOT a significant difference between the two groups
```

The final plot was taken into Adobe Illustrator to add significance
bars, remove the NA column, and make aesthetic changes.

```
#plot the composite graph:
girth_plot <- ggplot() +
  geom_violin(data = fish_profiles, aes(status, girth, colour = status, fill = status), trim = FALSE)  + #makes the violin plots
  geom_boxplot(data = fish_profiles, aes(status, girth, fill= status), width=0.2, colour = "white", outlier.color="white") + #overlays the boxplots over the violin plots
  scale_y_continuous(breaks = seq(2, 12, 2), limits = c(2,12), expand = c(0, 0)) + #sets the scaling parameters for the y axis
  scale_x_discrete(expand = expansion(add = c(0,1))) + #sets the scaling parameters for the x axis
  geom_segment(aes(x = 0, y = 2, xend = 0, yend = 12), lineend = "round", colour = "#1E1F17") + #redraws the y-axis
  labs(title = "girth of the fish", y="girth (mm)") + #defines the x and y axis labels
  scale_fill_manual(name = "status", values=c("#227266", "#D86631")) + #defines the fill aesthetics in the legend
  scale_colour_manual(name = "status", values=c("#227266", "#D86631")) + #defines the color aesthetics in the legend
  theme(plot.title = element_text(size = 12, hjust = 0.5),
        text = element_text(size = 16, colour = "#1E1F17", family = "sans"),
        axis.title.x = element_blank(),
        axis.title.y = element_text(vjust = 1.5),
        axis.line = element_blank(),
        axis.ticks.length = unit(2, "mm"),
        axis.ticks.x = element_blank(),
        axis.ticks = element_line(colour = "#1E1F17",lineend = "round"),
        panel.background = element_blank(),
        legend.key = element_blank(),
        legend.position = "none") #controls theme elements for the entire plot
girth_plot
```

# 4 Training & Associative Learning

## 4.1 Overview

A visual overview of all the trials fish needed to complete each of
the training phases (incl. habituation and associative learning). This
final plot was brought into Adobe Illustrator to rotate it 90 degrees,
remove the NA strip, and to make aesthetic changes, such as curving the
corners of the bars.

```
#first re-order the fish codes to the desired order and then reorder the levels of single & paired.
trials_pre_learning_FINAL$fish_code <- factor(trials_pre_learning_FINAL$fish_code, levels=c("V10", "V21", "V9", "V3", "V25", "V11", "V1", "V19", "V6", "V7", "V16", "V20", "P18", "P2", "P4", "P1", "P19", "P7", "P3", "P20", "P15", "P11", "P17", "P10"))
trials_pre_learning_FINAL$phase_learning <- factor(trials_pre_learning_FINAL$phase_learning, levels = c("3/3", "2/3", "1/3", "1well", "4well", "habituation"))

#define the strips which will be used to divide the single and paired data in the final plot.
status_strips <- strip_themed(background_x = elem_list_rect(fill = c("#227266", "#D86631"), colour = c("#227266", "#D86631")))
status_anno <- data.frame(x1 = c(0, 0), x2 = c(0, 0), 
                   y1 = c(0, 0), y2 = c(120, 0),
                   status = c("single", "paired"))
status_anno$status <- factor(status_anno$status, levels = c("single", "paired"))

#make the composite plot:
pre_learning_trials_overall_plot <- ggplot(data = trials_pre_learning_FINAL) +
  geom_bar(position = "stack", stat = "identity", aes(fill = phase_learning, y = trials, x = fish_code)) +
  facet_grid2(~status, scales = "free_x", switch = "x", strip = status_strips) +
  scale_y_continuous(breaks = seq(0, 120, 10), limits = c(0,120), expand = expansion(add = c(0, 0))) + #sets the scaling parameters for the y axis
  scale_x_discrete(expand = expansion(add = c(0,1))) + #sets the scaling parameters for the x axis
  geom_segment(data  = status_anno, aes(x = x1, y = y1, xend = x2, yend = y2), lineend = "round", colour = "#252625") + #redraws the y-axis
  geom_segment(aes(x = 0, y = 111, xend = 13, yend = 111), lineend = "round", linetype = "dotted",  colour = "#252625") +
  labs(title = "training trials overview", y="number of trials") + #defines the x and y axis labels
  scale_fill_manual(name = "phase", labels = c("3/3", "2/3", "1/3", "1 well", "4 well", "habituation"), values=c("#5A8B92", "#54AEB5", "#9DD9DB", "#C2503A", "#C79044", "#6B7D35")) + #defines the fill aesthetics in the legend
  scale_colour_manual(name = "phase", labels = c("3/3", "2/3", "1/3", "1 well", "4 well", "habituation"), values=c("#5A8B92", "#54AEB5", "#9DD9DB", "#C2503A", "#C79044", "#6B7D35")) + #defines the color aesthetics in the legend
  theme(plot.title = element_text(size = 12, hjust = 0.5),
        text = element_text(size = 16, colour = "#252625", family = "sans"),
        axis.text.x = element_blank(),
        axis.title.x = element_blank(),
        axis.title.y = element_text(vjust = 1.5),
        axis.line = element_blank(),
        axis.ticks.length = unit(2, "mm"),
        axis.ticks = element_line(colour = "#252625",lineend = "round"),
        axis.ticks.x = element_blank(),
        strip.text = element_text(face = "bold"),
        panel.background = element_blank(),
        panel.spacing.x = unit(0, "lines"),
        legend.key = element_blank(),
        legend.position = "right") #controls theme elements for the entire plot
pre_learning_trials_overall_plot
```

## 4.2 Stats & Plot

Determining whether coercive mating (single or paired) has an impact
on the total number of training trials fish needed in the experiment.
Normality of the data is first assessed using a Shapiro Wilk’s test and
visualized with a density plot. Summary statistics, wilcox test results
and final plots are shown below.

```
#test to see if the data are normally distributed:
#If p>0.05 then data ARE normal. If p<0.05 then data are NOT normal.
#p>0.05 so data ARE normal!
shapiro.test(fish_profiles$trials_prelearning)
```

```
## 
##  Shapiro-Wilk normality test
## 
## data:  fish_profiles$trials_prelearning
## W = 0.8403, p-value = 0.001452
```

```
ggdensity(fish_profiles$trials_prelearning)
```

```
#compute summary statistics per group (single vs paired):
group_by(fish_profiles, status) %>% summarise(count = n(), 
                                       median = median(trials_prelearning, na.rm = TRUE), 
                                       IQR = IQR(trials_prelearning, na.rm = TRUE))
```

```
## # A tibble: 3 × 4
##   status count median   IQR
##   <fct>  <int>  <dbl> <dbl>
## 1 single    12   51    11.2
## 2 paired    12   49.5  24.8
## 3 <NA>     288   NA    NA
```

```
#A non-parametric test is necessary since the data are non-normal.
wilcox.test(trials_prelearning ~ status, data = fish_profiles, exact = FALSE)
```

```
## 
##  Wilcoxon rank sum test with continuity correction
## 
## data:  trials_prelearning by status
## W = 74, p-value = 0.9301
## alternative hypothesis: true location shift is not equal to 0
```

```
#p>0.05 there is NOT a significant difference between the two groups
```

The final plot was taken into Adobe Illustrator to add significance
bars, remove the NA column, and make aesthetic changes.

```
#plot the composite graph:
trials_associative_plot <- ggplot() +
  geom_violin(data = fish_profiles, aes(status, trials_prelearning, colour = status, fill = status), trim = FALSE)  + #makes the violin plots
  geom_boxplot(data = fish_profiles, aes(status, trials_prelearning, fill= status), width=0.2, colour = "white", outlier.color="white") + #overlays the boxplots over the violin plots
  scale_y_continuous(breaks = seq(0, 140, 20), limits = c(0,140), expand = c(0, 0)) + #sets the scaling parameters for the y axis
  scale_x_discrete(expand = expansion(add = c(0,1))) + #sets the scaling parameters for the x axis
  geom_segment(aes(x = 0, y = 0, xend = 0, yend = 140), lineend = "round", colour = "#1E1F17") + #redraws the y-axis
  labs(title = "trials to complete learning", y="number of trials") + #defines the x and y axis labels
  scale_fill_manual(name = "status", values=c("#227266", "#D86631")) + #defines the fill aesthetics in the legend
  scale_colour_manual(name = "status", values=c("#227266", "#D86631")) + #defines the color aesthetics in the legend
  theme(plot.title = element_text(size = 12, hjust = 0.5),
        text = element_text(size = 16, colour = "#1E1F17", family = "sans"),
        axis.title.x = element_blank(),
        axis.title.y = element_text(vjust = 1.5),
        axis.line = element_blank(),
        axis.ticks.length = unit(2, "mm"),
        axis.ticks.x = element_blank(),
        axis.ticks = element_line(colour = "#1E1F17",lineend = "round"),
        panel.background = element_blank(),
        legend.key = element_blank(),
        legend.position = "none") #controls theme elements for the entire plot
trials_associative_plot
```

# 5 Spatial Learning

## 5.1 Success of the first disk push

Determining whether coercive mating (single or paired) had an impact
on the success of a fish’s first disk push (where non-choice is
incorrect).

```
#response variable: success of the first push (binomial; 1 = success, 0 = failed)
#potential predictors: status (single or paired), trial number in phase (1-36), the interaction between status and trial number, and fish identity as a random variable.
task_testing_success_push_glm1 <- glmer(success_push ~ status + trial_inphase + status:trial_inphase + (1|fish_code),
                             family= binomial, data = task_testing_FINAL)
summary(task_testing_success_push_glm1)
```

```
## Generalized linear mixed model fit by maximum likelihood (Laplace
##   Approximation) [glmerMod]
##  Family: binomial  ( logit )
## Formula: success_push ~ status + trial_inphase + status:trial_inphase +  
##     (1 | fish_code)
##    Data: task_testing_FINAL
## 
##      AIC      BIC   logLik deviance df.resid 
##    270.5    293.9   -130.2    260.5      787 
## 
## Scaled residuals: 
##     Min      1Q  Median      3Q     Max 
## -7.8449  0.1434  0.1711  0.2098  0.4536 
## 
## Random effects:
##  Groups    Name        Variance Std.Dev.
##  fish_code (Intercept) 0.1458   0.3819  
## Number of obs: 792, groups:  fish_code, 22
## 
## Fixed effects:
##                            Estimate Std. Error z value Pr(>|z|)    
## (Intercept)                 1.86444    0.40467   4.607 4.08e-06 ***
## statussingle                2.29002    0.81732   2.802  0.00508 ** 
## trial_inphase               0.06696    0.02396   2.794  0.00521 ** 
## statussingle:trial_inphase -0.09387    0.03844  -2.442  0.01461 *  
## ---
## Signif. codes:  0 '***' 0.001 '**' 0.01 '*' 0.05 '.' 0.1 ' ' 1
## 
## Correlation of Fixed Effects:
##             (Intr) sttssn trl_np
## statussingl -0.465              
## trial_inphs -0.739  0.371       
## sttssngl:t_  0.460 -0.840 -0.624
```

## 5.2 Whether fish make a choice

Determining whether coercive mating (single or paired) had an impact
on whether or not fish make a choice over the course of the phase.

```
#response variable: whether fish make a choice (binomial; 1 = yes, 0 = no)
#potential predictors: status (single or paired), trial number in phase (1-36), the interaction between status and trial number, and fish identity as a random variable.
task_testing_choice_push_glm1 <- glmer(choice_push ~ status + trial_inphase + status:trial_inphase + (1|fish_code),
                                        family= binomial, data = task_testing_FINAL)
summary(task_testing_choice_push_glm1)
```

```
## Generalized linear mixed model fit by maximum likelihood (Laplace
##   Approximation) [glmerMod]
##  Family: binomial  ( logit )
## Formula: choice_push ~ status + trial_inphase + status:trial_inphase +  
##     (1 | fish_code)
##    Data: task_testing_FINAL
## 
##      AIC      BIC   logLik deviance df.resid 
##    172.5    195.9    -81.3    162.5      787 
## 
## Scaled residuals: 
##      Min       1Q   Median       3Q      Max 
## -10.8177   0.0882   0.1173   0.1658   0.3660 
## 
## Random effects:
##  Groups    Name        Variance Std.Dev.
##  fish_code (Intercept) 0.4494   0.6703  
## Number of obs: 792, groups:  fish_code, 22
## 
## Fixed effects:
##                            Estimate Std. Error z value Pr(>|z|)    
## (Intercept)                 3.00280    0.58528   5.131 2.89e-07 ***
## statussingle                2.40654    1.33360   1.805   0.0711 .  
## trial_inphase               0.03057    0.02718   1.125   0.2606    
## statussingle:trial_inphase -0.06172    0.05687  -1.085   0.2778    
## ---
## Signif. codes:  0 '***' 0.001 '**' 0.01 '*' 0.05 '.' 0.1 ' ' 1
## 
## Correlation of Fixed Effects:
##             (Intr) sttssn trl_np
## statussingl -0.387              
## trial_inphs -0.715  0.317       
## sttssngl:t_  0.341 -0.866 -0.478
```

## 5.3 Whether fish who make a choice are successful

Determining whether coercive mating (single or paired) had an impact
on the success of a fish’s first disk push (where non-choice trials are
excluded).

```
#response variable: success of the first push when fish make a choice (binomial; 1 = success, 0 = failed)
#potential predictors: status (single or paired), trial number in phase (1-36), the interaction between status and trial number, and fish identity as a random variable.
task_testing_success_choice_push_glm1 <- glmer(success_choice_push ~ status + trial_inphase + status:trial_inphase + (1|fish_code),
                                       family= binomial, data = task_testing_FINAL)
```

```
## boundary (singular) fit: see help('isSingular')
```

```
summary(task_testing_success_choice_push_glm1)
```

```
## Generalized linear mixed model fit by maximum likelihood (Laplace
##   Approximation) [glmerMod]
##  Family: binomial  ( logit )
## Formula: success_choice_push ~ status + trial_inphase + status:trial_inphase +  
##     (1 | fish_code)
##    Data: task_testing_FINAL
## 
##      AIC      BIC   logLik deviance df.resid 
##    144.8    168.1    -67.4    134.8      769 
## 
## Scaled residuals: 
##      Min       1Q   Median       3Q      Max 
## -10.5998   0.0737   0.1236   0.1504   0.3527 
## 
## Random effects:
##  Groups    Name        Variance  Std.Dev. 
##  fish_code (Intercept) 3.778e-14 1.944e-07
## Number of obs: 774, groups:  fish_code, 22
## 
## Fixed effects:
##                            Estimate Std. Error z value Pr(>|z|)    
## (Intercept)                 1.91971    0.56021   3.427 0.000611 ***
## statussingle                2.56525    1.03656   2.475 0.013332 *  
## trial_inphase               0.16482    0.06128   2.690 0.007153 ** 
## statussingle:trial_inphase -0.18904    0.07180  -2.633 0.008470 ** 
## ---
## Signif. codes:  0 '***' 0.001 '**' 0.01 '*' 0.05 '.' 0.1 ' ' 1
## 
## Correlation of Fixed Effects:
##             (Intr) sttssn trl_np
## statussingl -0.540              
## trial_inphs -0.759  0.410       
## sttssngl:t_  0.648 -0.744 -0.853
## optimizer (Nelder_Mead) convergence code: 0 (OK)
## boundary (singular) fit: see help('isSingular')
```

## 5.4 Plot spatial learning curves

### 5.4.1 Success push

The learning curves for the success of the first disk push (where
non-choice is incorrect). This graph was then taken into Adobe
Illustrator to offset the points generated by geom\_count so that they do
not overlap the learning curves.

```
#Determine the marginal effect of status:trial_inphase on success_push according to our model (where NA values are NOT removed) and convert this to a data frame.
task_testing_success_push_glm1_fit <- effect('status*trial_inphase', task_testing_success_push_glm1, xlevels = 36, na.rm=FALSE) %>% as.data.frame()

#make a plot combining the results from our glm (using the fitting line generated above) and the distribution of the raw data (represented by geom_count):
#first reorder the levels of single & paired in the data frame
task_testing_success_push_glm1_fit$status <- factor(task_testing_success_push_glm1_fit$status, levels = c("single", "paired"))

#plot the composite graph:
task_testing_success_push_glm1_plot <- ggplot() +
  geom_line(data = task_testing_success_push_glm1_fit, aes(trial_inphase, fit, colour = status), lineend = "round", linewidth = 1.5)  + #makes the fitting lines
  geom_ribbon(data = task_testing_success_push_glm1_fit, aes(trial_inphase, NULL, ymin = lower, ymax = upper, fill = status), col = NA, alpha = 0.15,) + #makes the ribbons of fit around each fitting line
  geom_count(data = task_testing_FINAL, aes(x=trial_inphase, y= success_push, colour = status), position = ggstance::position_dodgev(height = 0.09), show.legend = FALSE) + #generate) + #generated points at 0 & 1 with sizes relative to the number of fish represented by that point
  scale_size_area() + #improves scaling of the points generated by geom_count
  scale_x_continuous(breaks=seq(2, 36, 2), limits = c(0, 37), expand =  c(0, 0)) + #defines the scaling parameters for the x axis
  scale_y_continuous(breaks = seq(0, 1, 0.5), limits = c(-0.1,1.1), expand = c(0, 0)) + #sets the scaling parameters for the y axis
  geom_segment(aes(x = 1, y = 0.5, xend = 36, yend = 0.5), lineend = "round", lty = 3, alpha = 0.3, colour = "#1E1F17") + #adds a horizontal line at 0.5
  geom_segment(aes(x = 1, y = 0.75, xend = 36, yend = 0.75), lineend = "round", lty = 3, alpha = 0.3, colour = "#1E1F17") + #adds a horizontal line at 0.75
  geom_segment(aes(x = 1, y = -0.1, xend = 36, yend = -0.1), lineend = "round", colour = "#1E1F17") + #redraws the y-axis
  geom_segment(aes(x = 0, y = 0, xend = 0, yend = 1), lineend = "round", colour = "#1E1F17") + #redraws the x-axis
  labs(title = "spatial learning: are fish successful in their first choice", subtitle = "where non-choice is treated as incorrect", x = "trial number", y="success of first choice") + #defines the x and y axis labels
  scale_fill_manual(name = "reproductive state", values=c("#227266", "#D86631")) + #defines the fill aesthetics in the legend
  scale_colour_manual(name = "reproductive state", values=c("#227266", "#D86631")) + #defines the color aesthetics in the legend
  theme(plot.title = element_text(size = 12, hjust = 0.5),
        plot.subtitle = element_text(size = 9, hjust = 0.5),
        text = element_text(size = 16, colour = "#1E1F17", family = "sans"),
        axis.title.x = element_text(vjust = -1),
        axis.title.y = element_text(vjust = 1.5),
        axis.line = element_blank(),
        axis.ticks.length = unit(2, "mm"),
        axis.ticks = element_line(colour = "#1E1F17",lineend = "round"),
        panel.background = element_blank(),
        legend.key = element_blank(),
        legend.position = "bottom") #controls theme elements for the entire plot
task_testing_success_push_glm1_plot
```

### 5.4.2 Choice push

The learning curves for whether or not fish make a choice over the
course of the phase. This graph was then taken into Adobe Illustrator to
offset the points generated by geom\_count so that they do not overlap
the learning curves.

```
#Determine the marginal effect of status:trial_inphase on choice_push according to our model (where NA values are NOT removed) and convert this to a data frame.
task_testing_choice_push_glm1_fit <- effect('status*trial_inphase', task_testing_choice_push_glm1, xlevels = 36, na.rm=FALSE) %>% as.data.frame()

#make a plot combining the results from our glm (using the fitting line generated above) and the distribution of the raw data (represented by geom_count):
#first reorder the levels of single & paired in the data frame
task_testing_choice_push_glm1_fit$status <- factor(task_testing_choice_push_glm1_fit$status, levels = c("single", "paired"))

#plot the composite graph:
task_testing_choice_push_glm1_plot <- ggplot() +
  geom_line(data = task_testing_choice_push_glm1_fit, aes(trial_inphase, fit, colour = status), lineend = "round", linewidth = 1.5)  + #makes the fitting lines
  geom_ribbon(data = task_testing_choice_push_glm1_fit, aes(trial_inphase, NULL, ymin = lower, ymax = upper, fill = status), col = NA, alpha = 0.15,) + #makes the ribbons of fit around each fitting line
  geom_count(data = task_testing_FINAL, aes(x=trial_inphase, y= choice_push, colour = status), position = ggstance::position_dodgev(height = 0.09), show.legend = FALSE) + #generated points at 0 & 1 with sizes relative to the number of fish represented by that point
  scale_size_area() + #improves scaling of the points generated by geom_count
  scale_x_continuous(breaks=seq(2, 36, 2), limits = c(0, 37), expand =  c(0, 0)) + #defines the scaling parameters for the x axis
  scale_y_continuous(breaks = seq(0, 1, 0.5), limits = c(-0.1,1.1), expand = c(0, 0)) + #sets the scaling parameters for the y axis
  geom_segment(aes(x = 1, y = 0.5, xend = 36, yend = 0.5), lineend = "round", lty = 3, alpha = 0.3, colour = "#1E1F17") + #adds a horizontal line at 0.5
  geom_segment(aes(x = 1, y = 0.75, xend = 36, yend = 0.75), lineend = "round", lty = 3, alpha = 0.3, colour = "#1E1F17") + #adds a horizontal line at 0.75
  geom_segment(aes(x = 1, y = -0.1, xend = 36, yend = -0.1), lineend = "round", colour = "#1E1F17") + #redraws the y-axis
  geom_segment(aes(x = 0, y = 0, xend = 0, yend = 1), lineend = "round", colour = "#1E1F17") + #redraws the x-axis
  labs(title = "spatial learning: do fish make a choice?", subtitle = "where choice is assessed whether correct or incorrect", x = "trial number", y="do fish make a choice") + #defines the x and y axis labels
  scale_fill_manual(name = "reproductive state", values=c("#227266", "#D86631")) + #defines the fill aesthetics in the legend
  scale_colour_manual(name = "reproductive state", values=c("#227266", "#D86631")) + #defines the color aesthetics in the legend
  theme(plot.title = element_text(size = 12, hjust = 0.5),
        plot.subtitle = element_text(size = 9, hjust = 0.5),
        text = element_text(size = 16, colour = "#1E1F17", family = "sans"),
        axis.title.x = element_text(vjust = -1),
        axis.title.y = element_text(vjust = 1.5),
        axis.line = element_blank(),
        axis.ticks.length = unit(2, "mm"),
        axis.ticks = element_line(colour = "#1E1F17",lineend = "round"),
        panel.background = element_blank(),
        legend.key = element_blank(),
        legend.position = "bottom") #controls theme elements for the entire plot
task_testing_choice_push_glm1_plot
```

### 5.4.3 Success choice push

The learning curves for the success of the first disk push only when
fish make a choice. This graph was then taken into Adobe Illustrator to
offset the points generated by geom\_count so that they do not overlap
the learning curves.

```
#Determine the marginal effect of status:trial_inphase on success_choice_push according to our model (where NA values are NOT removed) and convert this to a data frame.
task_testing_success_choice_push_glm1_fit <- effect('status*trial_inphase', task_testing_success_choice_push_glm1, xlevels = 33, na.rm=FALSE) %>% as.data.frame()

#make a plot combining the results from our glm (using the fitting line generated above) and the distribution of the raw data (represented by geom_count):
#first reorder the levels of single & paired in the data frame
task_testing_success_choice_push_glm1_fit$status <- factor(task_testing_success_choice_push_glm1_fit$status, levels = c("single", "paired"))

#plot the composite graph:
task_testing_success_choice_push_glm1_plot <- ggplot() +
  geom_line(data = task_testing_success_choice_push_glm1_fit, aes(trial_inphase, fit, colour = status), lineend = "round", linewidth = 1.5)  + #makes the fitting lines
  geom_ribbon(data = task_testing_success_choice_push_glm1_fit, aes(trial_inphase, NULL, ymin = lower, ymax = upper, fill = status), col = NA, alpha = 0.15,) + #makes the ribbons of fit around each fitting line
  geom_count(data = task_testing_FINAL, aes(x=trial_inphase, y= success_choice_push, colour = status), position = ggstance::position_dodgev(height = 0.09), show.legend = FALSE) + #generated points at 0 & 1 with sizes relative to the number of fish represented by that point
  scale_size_area() + #improves scaling of the points generated by geom_count
  scale_x_continuous(breaks=seq(2, 36, 2), limits = c(0, 37), expand =  c(0, 0)) + #defines the scaling parameters for the x axis
  scale_y_continuous(breaks = seq(0, 1, 0.5), limits = c(-0.1,1.1), expand = c(0, 0)) + #sets the scaling parameters for the y axis
  geom_segment(aes(x = 1, y = 0.5, xend = 36, yend = 0.5), lineend = "round", lty = 3, alpha = 0.3, colour = "#1E1F17") + #adds a horizontal line at 0.5
  geom_segment(aes(x = 1, y = 0.75, xend = 36, yend = 0.75), lineend = "round", lty = 3, alpha = 0.3, colour = "#1E1F17") + #adds a horizontal line at 0.75
  geom_segment(aes(x = 1, y = -0.1, xend = 36, yend = -0.1), lineend = "round", colour = "#1E1F17") + #redraws the y-axis
  geom_segment(aes(x = 0, y = 0, xend = 0, yend = 1), lineend = "round", colour = "#1E1F17") + #redraws the x-axis
  labs(title = "spatial learning: are fish who make a choice successful in their first choice?", subtitle = "where non-choice trials are excluded", x = "trial number", y="success of first push") + #defines the x and y axis labels
  scale_fill_manual(name = "reproductive state", values=c("#227266", "#D86631")) + #defines the fill aesthetics in the legend
  scale_colour_manual(name = "reproductive state", values=c("#227266", "#D86631")) + #defines the color aesthetics in the legend
  theme(plot.title = element_text(size = 12, hjust = 0.5),
        plot.subtitle = element_text(size = 9, hjust = 0.5),
        text = element_text(size = 16, colour = "#1E1F17", family = "sans"),
        axis.title.x = element_text(vjust = -1),
        axis.title.y = element_text(vjust = 1.5),
        axis.line = element_blank(),
        axis.ticks.length = unit(2, "mm"),
        axis.ticks = element_line(colour = "#1E1F17",lineend = "round"),
        panel.background = element_blank(),
        legend.key = element_blank(),
        legend.position = "bottom") #controls theme elements for the entire plot
task_testing_success_choice_push_glm1_plot
```

## 5.5 Plot Heatmaps

### 5.5.1 Fish performance

The heatmap generated of the raw data for each fish in each trial of
the spatial learning task. This graph was taken into Adobe Illustrator
to make aesthetic changes such as curving the edges of the squares,
recoloring the squares, and marking NA values based on the raw data.

```
task_testing_hm <- task_testing_repro %>% dplyr::select(fish_code, status, training_moment:choice_push, time_push_min:X15min)
#includes P10 & S16 for these supplemental figures

#first re-order the fish codes to the desired order and then reorder the levels of single & paired.
task_testing_FINAL$fish_code <- factor(task_testing_FINAL$fish_code, levels=c("V10", "V21", "V9", "V3", "V25", "V11", "V1", "V19", "V6", "V7", "V16", "V20", "P18", "P2", "P4", "P1", "P19", "P7", "P3", "P20", "P15", "P11", "P17", "P10"))
task_testing_FINAL$status <- factor(task_testing_FINAL$status, levels = c("single", "paired"))

#plot the heatmap
task_testing_success_time_plot <- ggplot() + 
  geom_tile(aes(trial_inphase, fish_code, fill= success_push), data = task_testing_hm, color = "white", lwd = 0.5, na.rm = TRUE) +
  facet_grid(vars(status), scales = "free") +
  scale_y_discrete(expand = expansion(add = c(0,1))) + #sets the scaling parameters for the y axis
  scale_x_continuous(breaks = seq(2, 36, 2), limits = c(0,37), expand = expansion(add = c(0, 0))) + #sets the scaling parameters for the x axis
  labs(title = "success of first push", x = "trial number", fill = "success of first disk push") + #defines the x and y axis labels
  scale_fill_stepsn(colors = c("#2C2D25", "#8FA338"),
                       values = c(0, 1), na.value = "white") +
  theme(plot.title = element_text(size = 12, hjust = 0.5),
        text = element_text(size = 16, colour = "#252625", family = "sans"),
        axis.title.x = element_text(),
        axis.title.y = element_blank(),
        axis.line = element_blank(),
        axis.ticks = element_blank(),
        strip.text = element_text(face = "bold"),
        panel.background = element_blank(),
        panel.spacing.x = unit(0, "lines"),
        legend.position = "bottom") #controls theme elements for the entire plot
task_testing_success_time_plot
```

### 5.5.2 Time to push

The heatmap generated based on the time it took fish to push a disk
in each trial of the spatial learning task. This graph was taken into
Adobe Illustrator to make aesthetic changes such as curving the edges of
the squares, recoloring the grid labels, and marking the NA values based
on the raw data.

```
#first re-order the fish codes to the desired order and then reorder the levels of single & paired.
task_testing_hm$fish_code <- factor(task_testing_hm$fish_code, levels=c("V10", "V21", "V9", "V3", "V25", "V11", "V1", "V19", "V6", "V7", "V16", "V20", "P18", "P2", "P4", "P1", "P19", "P7", "P3", "P20", "P15", "P11", "P17", "P10"))
task_testing_hm$status <- factor(task_testing_hm$status, levels = c("single", "paired"))

#plot the heatmap
task_testing_time_plot <- ggplot() + 
  geom_tile(aes(trial_inphase, fish_code, fill= time_push_min), data = task_testing_hm, color = "white", lwd = 0.5, na.rm = TRUE) +
  facet_grid(vars(status), scales = "free") +
  scale_y_discrete(expand = expansion(add = c(0,1))) + #sets the scaling parameters for the y axis
  scale_x_continuous(breaks = seq(2, 36, 2), limits = c(0,37), expand = expansion(add = c(0, 0))) + #sets the scaling parameters for the x axis
  labs(title = "speed of first push", x = "trial number", y= "fish", fill = "time until first disk push") + #defines the x and y axis labels
  scale_fill_gradientn(colors = c("#C1D376", "#C1D376", "#8FA338", "#6B7D35", "#E28479", "#C2503A", "#933822"),
                       values = scales::rescale(c(0, 0.5, 1, 5, 10, 15, max(task_testing_hm$time_push_min))), na.value = "#2C2D25") +
  theme(plot.title = element_text(size = 12, hjust = 0.5),
        text = element_text(size = 16, colour = "#252625", family = "sans"),
        axis.title.x = element_text(),
        axis.title.y = element_blank(),
        axis.line = element_blank(),
        axis.ticks = element_blank(),
        strip.text = element_text(face = "bold"),
        panel.background = element_blank(),
        panel.spacing.x = unit(0, "lines"),
        legend.position = "bottom") #controls theme elements for the entire plot
task_testing_time_plot
```

# 6 Reversal Learning

## 6.1 Success of the first disk push

Determining whether coercive mating (single or paired) had an impact
on the success of a fish’s first disk push (where non-choice is
incorrect).

```
#response variable: success of the first push (binomial; 1 = success, 0 = failed)
#potential predictors: status (single or paired), trial number in phase (1-36), the interaction between status and trial number, and fish identity as a random variable.
reversal_learning_success_push_glm1 <- glmer(success_push ~ status + trial_inphase + status:trial_inphase + (1|fish_code),
                           family= binomial, data = reversal_learning_FINAL)
summary(reversal_learning_success_push_glm1)
```

```
## Generalized linear mixed model fit by maximum likelihood (Laplace
##   Approximation) [glmerMod]
##  Family: binomial  ( logit )
## Formula: success_push ~ status + trial_inphase + status:trial_inphase +  
##     (1 | fish_code)
##    Data: reversal_learning_FINAL
## 
##      AIC      BIC   logLik deviance df.resid 
##    587.7    611.1   -288.9    577.7      787 
## 
## Scaled residuals: 
##     Min      1Q  Median      3Q     Max 
## -7.2208  0.1515  0.2680  0.4508  0.9831 
## 
## Random effects:
##  Groups    Name        Variance Std.Dev.
##  fish_code (Intercept) 0.1531   0.3913  
## Number of obs: 792, groups:  fish_code, 22
## 
## Fixed effects:
##                            Estimate Std. Error z value Pr(>|z|)    
## (Intercept)                 0.24593    0.29454   0.835    0.404    
## statussingle               -0.09901    0.40295  -0.246    0.806    
## trial_inphase               0.11826    0.01988   5.949 2.69e-09 ***
## statussingle:trial_inphase -0.02257    0.02550  -0.885    0.376    
## ---
## Signif. codes:  0 '***' 0.001 '**' 0.01 '*' 0.05 '.' 0.1 ' ' 1
## 
## Correlation of Fixed Effects:
##             (Intr) sttssn trl_np
## statussingl -0.730              
## trial_inphs -0.728  0.534       
## sttssngl:t_  0.570 -0.734 -0.775
```

## 6.2 Whether fish make a choice

Determining whether coercive mating (single or paired) had an impact
on whether or not fish make a choice over the course of the phase.

```
#response variable: whether fish make a choice (binomial; 1 = yes, 0 = no)
#potential predictors: status (single or paired), trial number in phase (1-36), the interaction between status and trial number, and fish identity as a random variable.
reversal_learning_choice_push_glm1 <- glmer(choice_push ~ status + trial_inphase + status:trial_inphase + (1|fish_code),
                                       family= binomial, data = reversal_learning_FINAL)
summary(reversal_learning_choice_push_glm1)
```

```
## Generalized linear mixed model fit by maximum likelihood (Laplace
##   Approximation) [glmerMod]
##  Family: binomial  ( logit )
## Formula: choice_push ~ status + trial_inphase + status:trial_inphase +  
##     (1 | fish_code)
##    Data: reversal_learning_FINAL
## 
##      AIC      BIC   logLik deviance df.resid 
##    130.2    153.6    -60.1    120.2      787 
## 
## Scaled residuals: 
##      Min       1Q   Median       3Q      Max 
## -15.1362   0.0609   0.0644   0.1137   0.5665 
## 
## Random effects:
##  Groups    Name        Variance Std.Dev.
##  fish_code (Intercept) 1.782    1.335   
## Number of obs: 792, groups:  fish_code, 22
## 
## Fixed effects:
##                             Estimate Std. Error z value Pr(>|z|)    
## (Intercept)                 5.230977   1.227333   4.262 2.03e-05 ***
## statussingle               -1.727045   1.353539  -1.276    0.202    
## trial_inphase               0.004749   0.048737   0.097    0.922    
## statussingle:trial_inphase  0.067687   0.062091   1.090    0.276    
## ---
## Signif. codes:  0 '***' 0.001 '**' 0.01 '*' 0.05 '.' 0.1 ' ' 1
## 
## Correlation of Fixed Effects:
##             (Intr) sttssn trl_np
## statussingl -0.767              
## trial_inphs -0.715  0.648       
## sttssngl:t_  0.573 -0.720 -0.785
```

## 6.3 Whether fish who make a choice are successful

Determining whether coercive mating (single or paired) had an impact
on the success of a fish’s first disk push (where non-choice trials are
excluded).

```
#response variable: success of the first push when fish make a choice (binomial; 1 = success, 0 = failed)
#potential predictors: status (single or paired), trial number in phase (1-36), the interaction between status and trial number, and fish identity as a random variable.
reversal_learning_success_choice_push_glm1 <- glmer(success_choice_push ~ status + trial_inphase + status:trial_inphase + (1|fish_code),
                                               family= binomial, data = reversal_learning_FINAL)
summary(reversal_learning_success_choice_push_glm1)
```

```
## Generalized linear mixed model fit by maximum likelihood (Laplace
##   Approximation) [glmerMod]
##  Family: binomial  ( logit )
## Formula: success_choice_push ~ status + trial_inphase + status:trial_inphase +  
##     (1 | fish_code)
##    Data: reversal_learning_FINAL
## 
##      AIC      BIC   logLik deviance df.resid 
##    543.4    566.7   -266.7    533.4      775 
## 
## Scaled residuals: 
##     Min      1Q  Median      3Q     Max 
## -8.7710  0.1422  0.2525  0.4299  0.9081 
## 
## Random effects:
##  Groups    Name        Variance Std.Dev.
##  fish_code (Intercept) 0.0988   0.3143  
## Number of obs: 780, groups:  fish_code, 22
## 
## Fixed effects:
##                            Estimate Std. Error z value Pr(>|z|)    
## (Intercept)                 0.20611    0.29216   0.705    0.481    
## statussingle                0.03621    0.40166   0.090    0.928    
## trial_inphase               0.12969    0.02169   5.979 2.25e-09 ***
## statussingle:trial_inphase -0.03281    0.02749  -1.193    0.233    
## ---
## Signif. codes:  0 '***' 0.001 '**' 0.01 '*' 0.05 '.' 0.1 ' ' 1
## 
## Correlation of Fixed Effects:
##             (Intr) sttssn trl_np
## statussingl -0.726              
## trial_inphs -0.747  0.545       
## sttssngl:t_  0.592 -0.755 -0.785
```

## 6.4 Plot spatial learning curves

### 6.4.1 Success push

The learning curves for the success of the first disk push (where
non-choice is incorrect). This graph was then taken into Adobe
Illustrator to offset the points generated by geom\_count so that they do
not overlap the learning curves.

```
#Determine the marginal effect of status:trial_inphase on success_push according to our model (where NA values are NOT removed) and convert this to a data frame.
reversal_learning_success_push_glm1_fit <- effect('status*trial_inphase', reversal_learning_success_push_glm1, xlevels = 36, na.rm=TRUE) %>% as.data.frame()

#make a plot combining the results from our glm (using the fitting line generated above) and the distribution of the raw data (represented by geom_count):
#first reorder the levels of single & paired in the data frame
reversal_learning_success_push_glm1_fit$status <- factor(reversal_learning_success_push_glm1_fit$status, levels = c("single", "paired"))

#plot the composite graph:
reversal_learning_success_push_glm1_plot <- ggplot() +
  geom_line(data = reversal_learning_success_push_glm1_fit, aes(trial_inphase, fit, colour = status), lineend = "round", linewidth = 1.5)  + #makes the fitting lines
  geom_ribbon(data = reversal_learning_success_push_glm1_fit, aes(trial_inphase, NULL, ymin = lower, ymax = upper, fill = status), col = NA, alpha = 0.15,) + #makes the ribbons of fit around each fitting line
  geom_count(data = reversal_learning_FINAL, aes(x=trial_inphase, y= success_push, colour = status), position = ggstance::position_dodgev(height = 0.09), show.legend = FALSE) + #generated points at 0 & 1 with sizes relative to the number of fish represented by that point
  scale_size_area() + #improves scaling of the points generated by geom_count
  scale_x_continuous(breaks=seq(2, 36, 2), limits = c(0, 37), expand =  c(0, 0)) + #defines the scaling parameters for the x axis
  scale_y_continuous(breaks = seq(0, 1, 0.5), limits = c(-0.1,1.1), expand = c(0, 0)) + #sets the scaling parameters for the y axis
  geom_segment(aes(x = 1, y = 0.5, xend = 36, yend = 0.5), lineend = "round", lty = 3, alpha = 0.3, colour = "#1E1F17") + #adds a horizontal line at 0.5
  geom_segment(aes(x = 1, y = 0.75, xend = 36, yend = 0.75), lineend = "round", lty = 3, alpha = 0.3, colour = "#1E1F17") + #adds a horizontal line at 0.75
  geom_segment(aes(x = 1, y = -0.1, xend = 36, yend = -0.1), lineend = "round", colour = "#1E1F17") + #redraws the y-axis
  geom_segment(aes(x = 0, y = 0, xend = 0, yend = 1), lineend = "round", colour = "#1E1F17") + #redraws the x-axis
  labs(title = "reversal learning", subtitle = "where non-choice trials are failures", x = "trial #", y="success of first push") + #defines the x and y axis labels
  scale_fill_manual(name = "reproductive state", values=c("#227266", "#D86631")) + #defines the fill aesthetics in the legend
  scale_colour_manual(name = "reproductive state", values=c("#227266", "#D86631")) + #defines the color aesthetics in the legend
  theme(plot.title = element_text(size = 12, hjust = 0.5),
        plot.subtitle = element_text(size = 9, hjust = 0.5),
        text = element_text(size = 16, colour = "#1E1F17", family = "sans"),
        axis.title.x = element_text(vjust = -1),
        axis.title.y = element_text(vjust = 1.5),
        axis.line = element_blank(),
        axis.ticks.length = unit(2, "mm"),
        axis.ticks = element_line(colour = "#1E1F17",lineend = "round"),
        panel.background = element_blank(),
        legend.key = element_blank(),
        legend.position = "bottom") #controls theme elements for the entire plot
reversal_learning_success_push_glm1_plot
```

### 6.4.2 Choice push

The learning curves for whether or not fish make a choice over the
course of the phase. This graph was then taken into Adobe Illustrator to
offset the points generated by geom\_count so that they do not overlap
the learning curves.

```
#Determine the marginal effect of status:trial_inphase on choice_push according to our model (where NA values are NOT removed) and convert this to a data frame.
reversal_learning_choice_push_glm1_fit <- effect('status*trial_inphase', reversal_learning_choice_push_glm1, xlevels = 36, na.rm=FALSE) %>% as.data.frame()

#make a plot combining the results from our glm (using the fitting line generated above) and the distribution of the raw data (represented by geom_count):
#first reorder the levels of single & paired in the data frame
reversal_learning_choice_push_glm1_fit$status <- factor(reversal_learning_choice_push_glm1_fit$status, levels = c("single", "paired"))

#plot the composite graph:
reversal_learning_choice_push_glm1_plot <- ggplot() +
  geom_line(data = reversal_learning_choice_push_glm1_fit, aes(trial_inphase, fit, colour = status), lineend = "round", linewidth = 1.5)  + #makes the fitting lines
  geom_ribbon(data = reversal_learning_choice_push_glm1_fit, aes(trial_inphase, NULL, ymin = lower, ymax = upper, fill = status), col = NA, alpha = 0.15,) + #makes the ribbons of fit around each fitting line
  geom_count(data = reversal_learning_FINAL, aes(x=trial_inphase, y= choice_push, colour = status), position = ggstance::position_dodgev(height = 0.09), show.legend = FALSE) + #generated points at 0 & 1 with sizes relative to the number of fish represented by that point
  scale_size_area() + #improves scaling of the points generated by geom_count
  scale_x_continuous(breaks=seq(2, 36, 2), limits = c(0, 37), expand =  c(0, 0)) + #defines the scaling parameters for the x axis
  scale_y_continuous(breaks = seq(0, 1, 0.5), limits = c(-0.1,1.1), expand = c(0, 0)) + #sets the scaling parameters for the y axis
  geom_segment(aes(x = 1, y = 0.5, xend = 36, yend = 0.5), lineend = "round", lty = 3, alpha = 0.3, colour = "#1E1F17") + #adds a horizontal line at 0.5
  geom_segment(aes(x = 1, y = 0.75, xend = 36, yend = 0.75), lineend = "round", lty = 3, alpha = 0.3, colour = "#1E1F17") + #adds a horizontal line at 0.75
  geom_segment(aes(x = 1, y = -0.1, xend = 36, yend = -0.1), lineend = "round", colour = "#1E1F17") + #redraws the y-axis
  geom_segment(aes(x = 0, y = 0, xend = 0, yend = 1), lineend = "round", colour = "#1E1F17") + #redraws the x-axis
  labs(title = "reversal learning: do fish make a choice?", subtitle = "where choice is assessed whether correct or incorrect", x = "trial #", y="do fish make a choice") + #defines the x and y axis labels
  scale_fill_manual(name = "reproductive state", values=c("#227266", "#D86631")) + #defines the fill aesthetics in the legend
  scale_colour_manual(name = "reproductive state", values=c("#227266", "#D86631")) + #defines the color aesthetics in the legend
  theme(plot.title = element_text(size = 12, hjust = 0.5),
        plot.subtitle = element_text(size = 9, hjust = 0.5),
        text = element_text(size = 16, colour = "#1E1F17", family = "sans"),
        axis.title.x = element_text(vjust = -1),
        axis.title.y = element_text(vjust = 1.5),
        axis.line = element_blank(),
        axis.ticks.length = unit(2, "mm"),
        axis.ticks = element_line(colour = "#1E1F17",lineend = "round"),
        panel.background = element_blank(),
        legend.key = element_blank(),
        legend.position = "bottom") #controls theme elements for the entire plot
reversal_learning_choice_push_glm1_plot
```

### 6.4.3 Success choice push

The learning curves for the success of the first disk push only when
fish make a choice. This graph was then taken into Adobe Illustrator to
offset the points generated by geom\_count so that they do not overlap
the learning curves.

```
#Determine the marginal effect of status:trial_inphase on success_choice_push according to our model (where NA values are NOT removed) and convert this to a data frame.
reversal_learning_success_choice_push_glm1_fit <- effect('status*trial_inphase', reversal_learning_success_choice_push_glm1, xlevels = 36, na.rm=FALSE) %>% as.data.frame()

#make a plot combining the results from our glm (using the fitting line generated above) and the distribution of the raw data (represented by geom_count):
#first reorder the levels of single & paired in the data frame
reversal_learning_success_choice_push_glm1_fit$status <- factor(reversal_learning_success_choice_push_glm1_fit$status, levels = c("single", "paired"))

#plot the composite graph:
reversal_learning_success_choice_push_glm1_plot <- ggplot() +
  geom_line(data = reversal_learning_success_choice_push_glm1_fit, aes(trial_inphase, fit, colour = status), lineend = "round", linewidth = 1.5)  + #makes the fitting lines
  geom_ribbon(data = reversal_learning_success_choice_push_glm1_fit, aes(trial_inphase, NULL, ymin = lower, ymax = upper, fill = status), col = NA, alpha = 0.15,) + #makes the ribbons of fit around each fitting line
  geom_count(data = reversal_learning_FINAL, aes(x=trial_inphase, y= success_choice_push, colour = status), position = ggstance::position_dodgev(height = 0.09), show.legend = FALSE) + #generated points at 0 & 1 with sizes relative to the number of fish represented by that point
  scale_size_area() + #improves scaling of the points generated by geom_count
  scale_x_continuous(breaks=seq(2, 36, 2), limits = c(0, 37), expand =  c(0, 0)) + #defines the scaling parameters for the x axis
  scale_y_continuous(breaks = seq(0, 1, 0.5), limits = c(-0.1,1.1), expand = c(0, 0)) + #sets the scaling parameters for the y axis
  geom_segment(aes(x = 1, y = 0.5, xend = 36, yend = 0.5), lineend = "round", lty = 3, alpha = 0.3, colour = "#1E1F17") + #adds a horizontal line at 0.5
  geom_segment(aes(x = 1, y = 0.75, xend = 36, yend = 0.75), lineend = "round", lty = 3, alpha = 0.3, colour = "#1E1F17") + #adds a horizontal line at 0.75
  geom_segment(aes(x = 1, y = -0.1, xend = 36, yend = -0.1), lineend = "round", colour = "#1E1F17") + #redraws the y-axis
  geom_segment(aes(x = 0, y = 0, xend = 0, yend = 1), lineend = "round", colour = "#1E1F17") + #redraws the x-axis
  labs(title = "reversal learning: are fish who make a choice successful in their first choice?", subtitle = "where non-choice trials are excluded", x = "trial #", y="success of first push") + #defines the x and y axis labels
  scale_fill_manual(name = "reproductive state", values=c("#227266", "#D86631")) + #defines the fill aesthetics in the legend
  scale_colour_manual(name = "reproductive state", values=c("#227266", "#D86631")) + #defines the color aesthetics in the legend
  theme(plot.title = element_text(size = 12, hjust = 0.5),
        plot.subtitle = element_text(size = 9, hjust = 0.5),
        text = element_text(size = 16, colour = "#1E1F17", family = "sans"),
        axis.title.x = element_text(vjust = -1),
        axis.title.y = element_text(vjust = 1.5),
        axis.line = element_blank(),
        axis.ticks.length = unit(2, "mm"),
        axis.ticks = element_line(colour = "#1E1F17",lineend = "round"),
        panel.background = element_blank(),
        legend.key = element_blank(),
        legend.position = "bottom") #controls theme elements for the entire plot
reversal_learning_success_choice_push_glm1_plot
```

## 6.5 Plot Heatmaps

### 6.5.1 Fish performance

The heatmap generated of the raw data for each fish in each trial of
the reversal learning task. This graph was taken into Adobe Illustrator
to make aesthetic changes such as curving the edges of the squares,
recoloring the squares, and marking NA values based on the raw data.

```
reversal_learning_hm <- reversal_learning_repro %>% dplyr::select(fish_code, status, training_moment:choice_push, time_push_min:X15min)
#includes P10 & S16 for these supplemental figures

#first re-order the fish codes to the desired order and then reorder the levels of single & paired.
reversal_learning_hm$fish_code <- factor(reversal_learning_hm$fish_code, levels=c("V10", "V21", "V9", "V3", "V25", "V11", "V1", "V19", "V6", "V7", "V16", "V20", "P18", "P2", "P4", "P1", "P19", "P7", "P3", "P20", "P15", "P11", "P17", "P10"))
reversal_learning_hm$status <- factor(reversal_learning_hm$status, levels = c("single", "paired"))

#plot the heatmap
reversal_learning_success_time_plot <- ggplot() + 
  geom_tile(aes(trial_inphase, fish_code, fill= success_push), data = reversal_learning_hm, color = "white", lwd = 0.5, na.rm = TRUE) +
  facet_grid(vars(status), scales = "free") +
  scale_y_discrete(expand = expansion(add = c(0,1))) + #sets the scaling parameters for the y axis
  scale_x_continuous(breaks = seq(2, 36, 2), limits = c(0,37), expand = expansion(add = c(0, 0))) + #sets the scaling parameters for the x axis
  labs(title = "success of first push - reversal", x = "trial number", fill = "success of first disk push") + #defines the x and y axis labels
  scale_fill_stepsn(colors = c("#2C2D25", "#8FA338"),
                    values = c(0, 1), na.value = "white") +
  theme(plot.title = element_text(size = 12, hjust = 0.5),
        text = element_text(size = 16, colour = "#252625", family = "sans"),
        axis.title.x = element_text(),
        axis.title.y = element_blank(),
        axis.line = element_blank(),
        axis.ticks = element_blank(),
        strip.text = element_text(face = "bold"),
        panel.background = element_blank(),
        panel.spacing.x = unit(0, "lines"),
        legend.position = "bottom") #controls theme elements for the entire plot
reversal_learning_success_time_plot
```

### 6.5.2 Time to push

The heatmap generated based on the time it took fish to push a disk
in each trial of the reversal learning task. This graph was taken into
Adobe Illustrator to make aesthetic changes such as curving the edges of
the squares, recoloring the grid labels, and marking the NA values based
on the raw data.

```
#first re-order the fish codes to the desired order and then reorder the levels of single & paired.
reversal_learning_hm$fish_code <- factor(reversal_learning_hm$fish_code, levels=c("V10", "V21", "V9", "V3", "V25", "V11", "V1", "V19", "V6", "V7", "V16", "V20", "P18", "P2", "P4", "P1", "P19", "P7", "P3", "P20", "P15", "P11", "P17", "P10"))
reversal_learning_hm$status <- factor(reversal_learning_hm$status, levels = c("single", "paired"))

#plot the heatmap
reversal_learning_time_plot <- ggplot() + 
  geom_tile(aes(trial_inphase, fish_code, fill= time_push_min), data = reversal_learning_hm, color = "white", lwd = 0.5, na.rm = TRUE) +
  facet_grid(vars(status), scales = "free") +
  scale_y_discrete(expand = expansion(add = c(0,1))) + #sets the scaling parameters for the y axis
  scale_x_continuous(breaks = seq(2, 36, 2), limits = c(0,37), expand = expansion(add = c(0, 0))) + #sets the scaling parameters for the x axis
  labs(title = "speed of first push - reversal", x = "trial number", y= "fish", fill = "time until first disk push") + #defines the x and y axis labels
  scale_fill_gradientn(colors = c("#C1D376", "#C1D376", "#8FA338", "#6B7D35", "#E28479", "#C2503A", "#933822"),
                       values = scales::rescale(c(0, 0.5, 1, 5, 10, 15, max(reversal_learning_hm$time_push_min))), na.value = "#252625") +
  theme(plot.title = element_text(size = 12, hjust = 0.5),
        text = element_text(size = 16, colour = "#252625", family = "sans"),
        axis.title.x = element_text(),
        axis.title.y = element_blank(),
        axis.line = element_blank(),
        axis.ticks = element_blank(),
        strip.text = element_text(face = "bold"),
        panel.background = element_blank(),
        panel.spacing.x = unit(0, "lines"),
        legend.position = "bottom") #controls theme elements for the entire plot
reversal_learning_time_plot
```
