# Supplementary figures and images for: Coercive mating has no impact on spatial learning, cognitive flexibility, and fecundity in female porthole livebearers (Poeciliopsis gracilis)
Source: J Fish Biol. 2024 Feb 25;107(4):1106–21. doi: 10.1111/jfb.15696 (PMC12536062; doi:10.1111/jfb.15696)

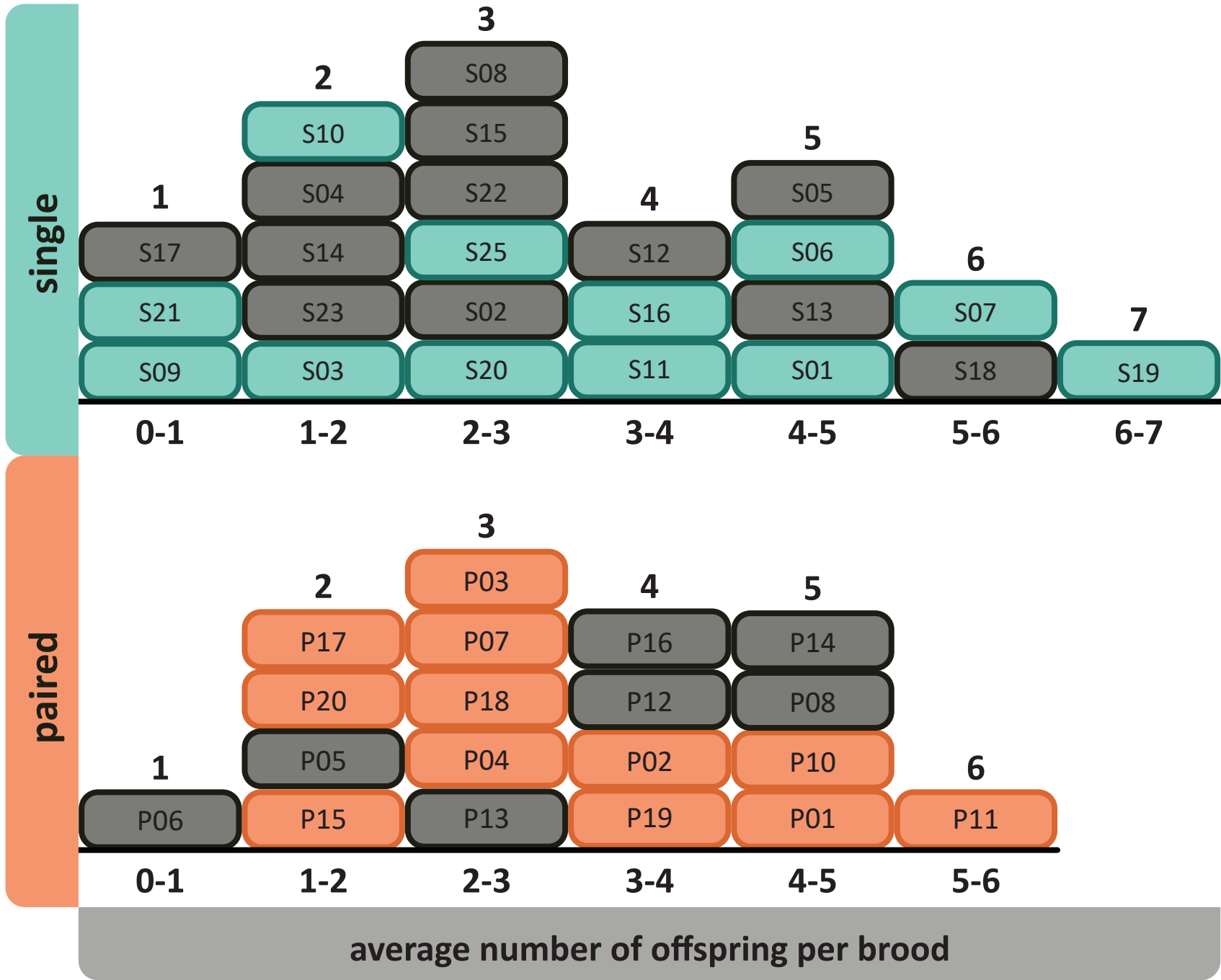

Supplement: Supplementary file 3 — FIGURE S1. A schematic of the ranks generated for fish selection, based on the average number of offspring per brood for each fish. The ranks are given by the number listed at the top of each stack. Ranges running along the y axis indicate the average number of offspring per brood for fish in those ranks, where rank 1 has a range of 0 to 1 but not including 1, rank 2 has a range of 1 to 2 but not including 2 and so on. Fish were ranked within their assigned treatment group (single, above; paired, below). Fish which were not selected for the experiment are shown in gray and selected fish are shown in teal and orange for single and paired fish, respectively. [file JFB-107-1106-s004.pdf]

**(a)**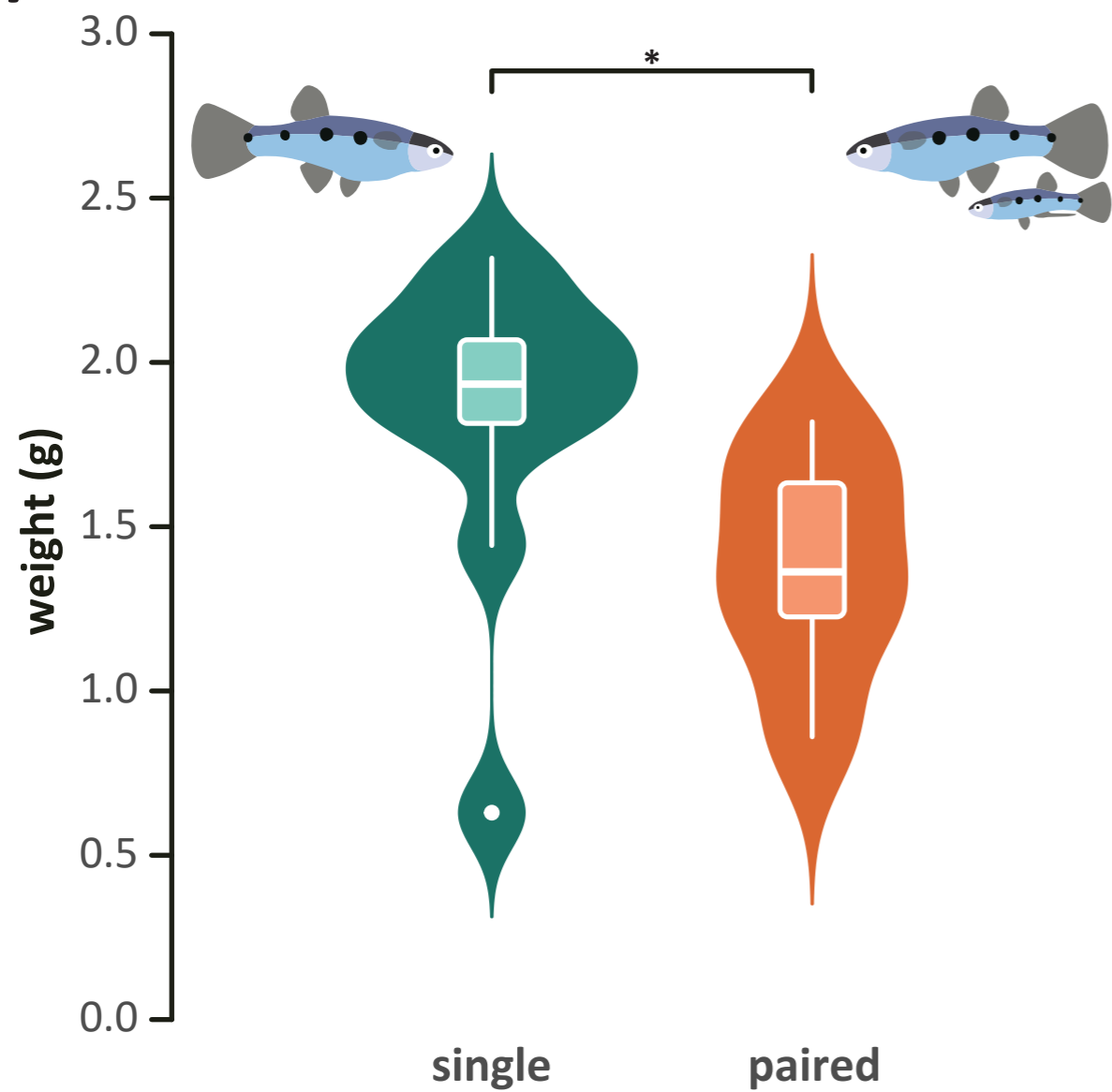**(b)**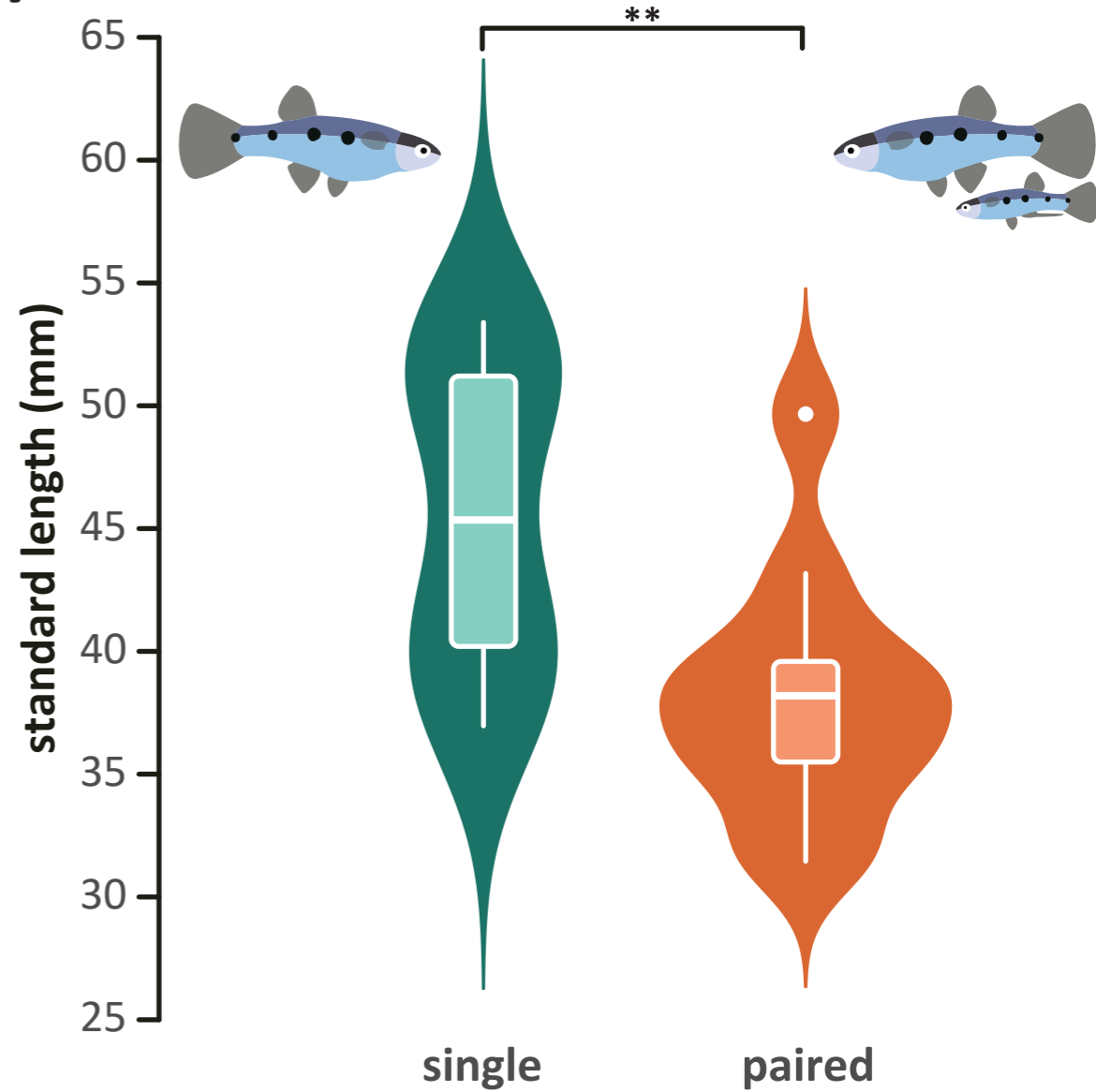**(c)**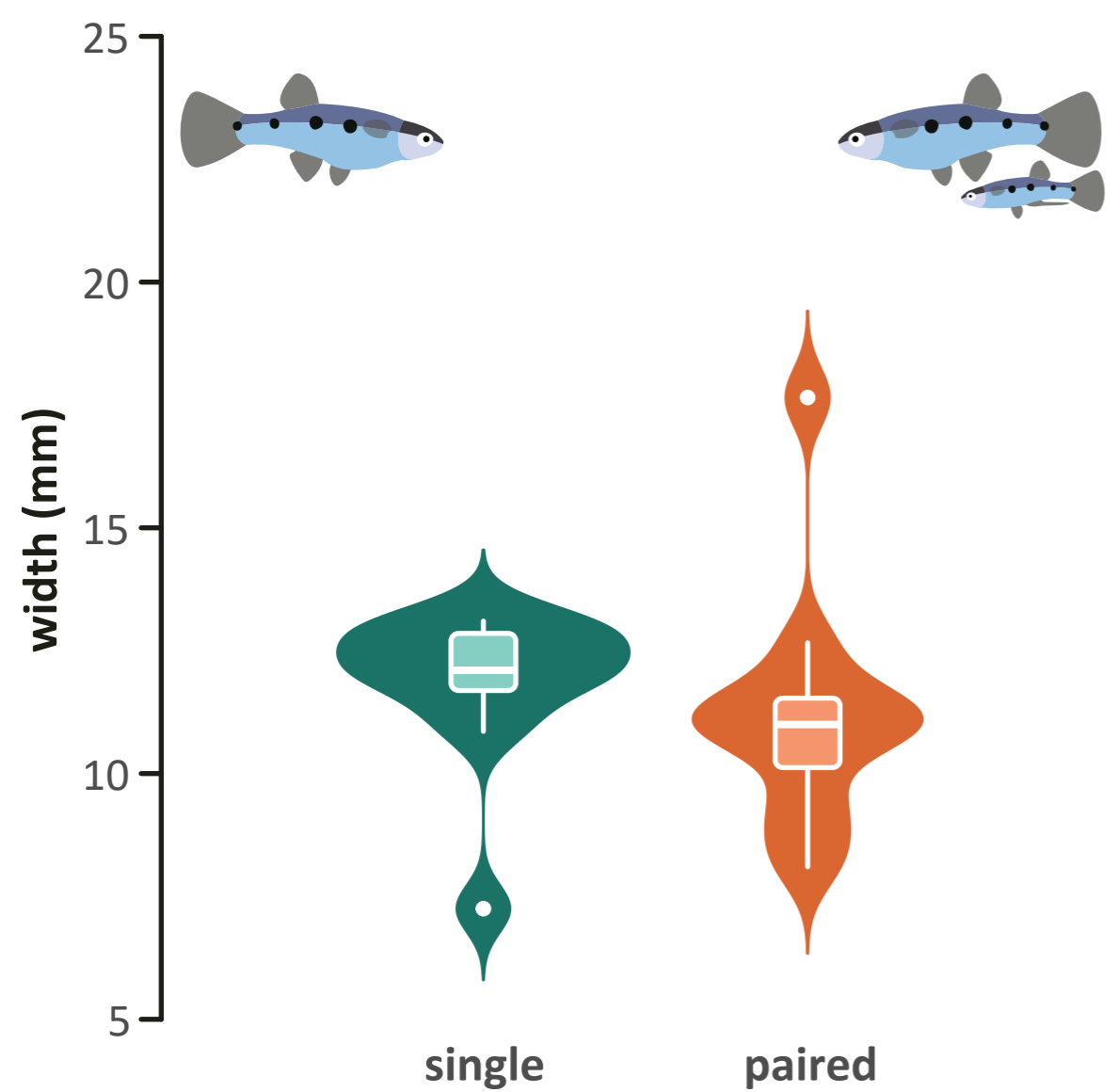**(d)**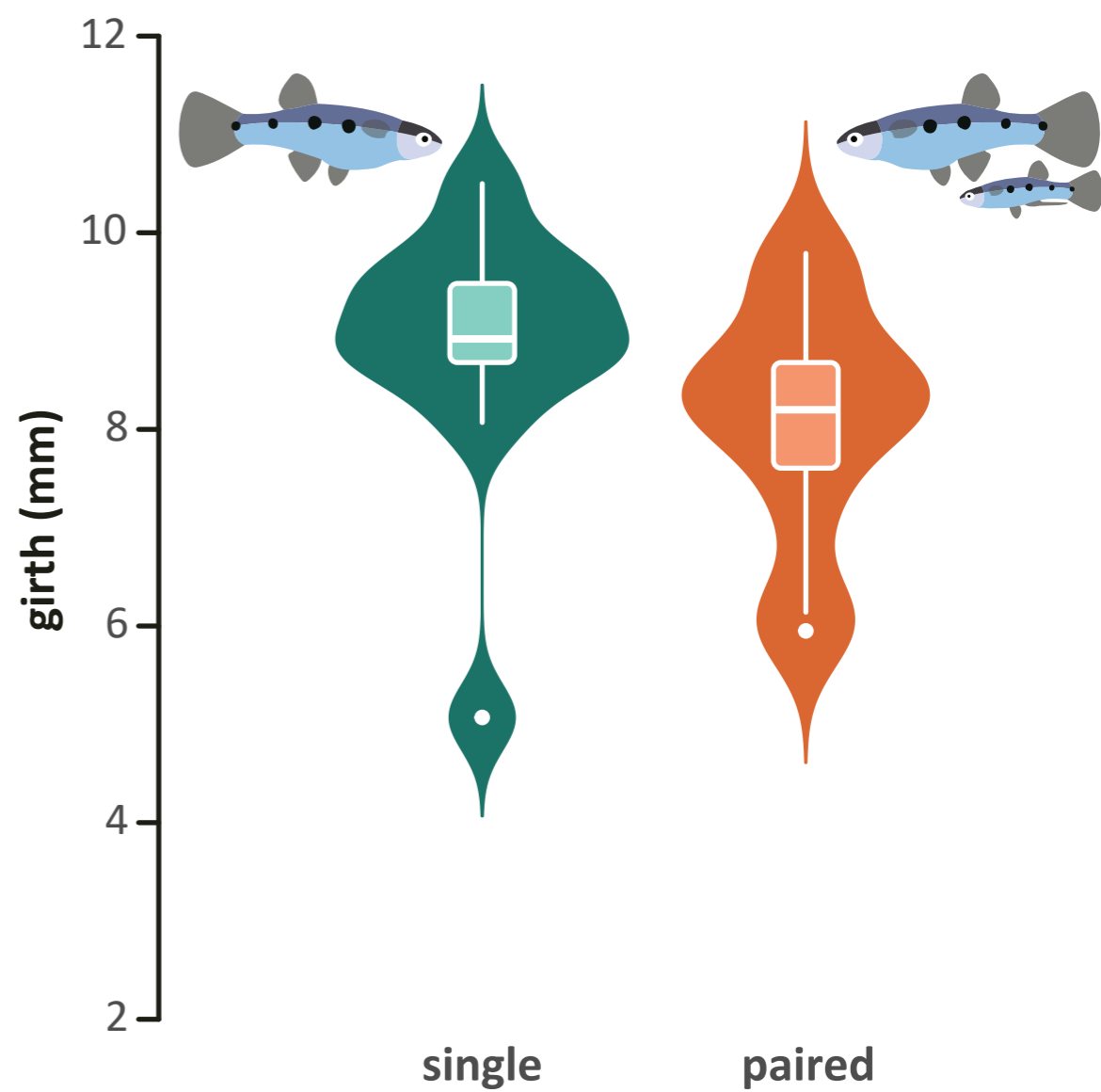

Supplement: Supplementary file 4 — FIGURE S2. Morphological characteristics of the fish in this study as measured at the end of the experiment. (a) Weight of the fish in grams (t = 2.6561, df = 18.04, p value = 0.01606). (b) Standard length of the fish in millimeters (W = 101, p value = 0.006634). (c) Width of the fish in millimeters at the widest point (W = 88, p value = 0.07589). (d) Girth of the fish in millimeters at the widest point (W = 87, p value = 0.08795). [file JFB-107-1106-s003.pdf]

**(a)**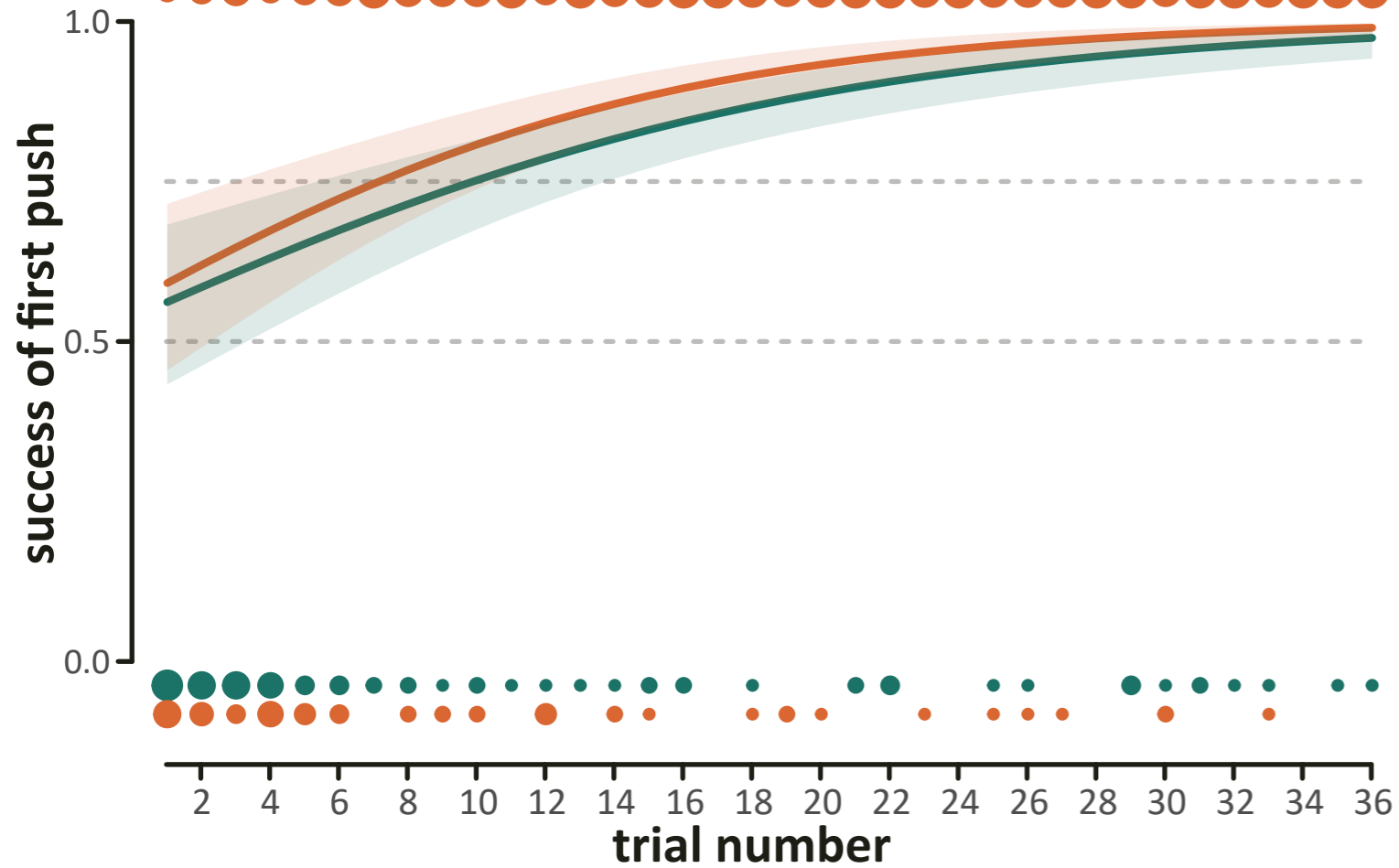**(b)**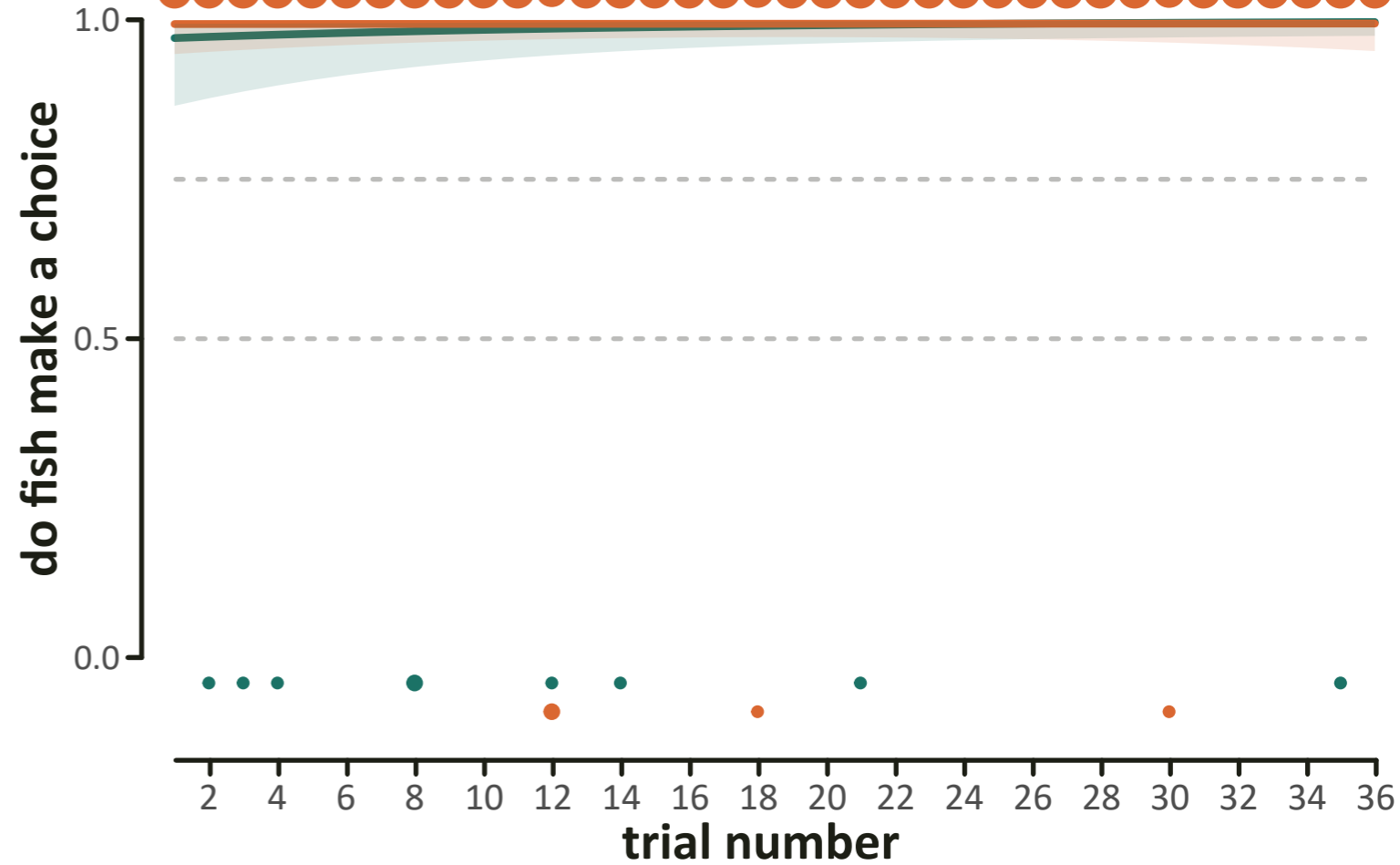

number of fish: 2.5, 5, 7.5, 10  
treatment group: single, paired

Supplement: Supplementary file 6 — FIGURE S4. Reversal learning curves as generated by our generalized linear mixed models. Lines indicate the model fit of the effect of the interaction variable (treatment group [single] × trial number) with the lower and upper bounds of the interquartile range. Circles above and below the axes indicate the number of fish from each group who succeed (1) or fail (0) in each trial, where the area of the circle increases in proportion to the number of fish from 0 to 11. (a) The success of the first disk push, where non‐choice trials are treated as failures. (b) Whether or not fish make a choice in each trial, where any disk push is a success and non‐choice is a fail. [file JFB-107-1106-s002.pdf]
