# Supplementary material for: Coercive mating has no impact on spatial learning, cognitive flexibility, and fecundity in female porthole livebearers (Poeciliopsis gracilis)
Source: J Fish Biol. 2024 Feb 25;107(4):1106–21. doi: 10.1111/jfb.15696 (PMC12536062; doi:10.1111/jfb.15696)
Supplement: Supplementary file 5 — FIGURE S3. Heat maps representing the raw data from the spatial and reversal learning tasks. Fish excluded from the final analyses are marked with an “X" next to their identity code. (a) The success of each fish on their first disk push during the spatial learning task, where all failures (0) are shown in black, non‐choice trials are shown in gray, and successes (1) are shown in teal or orange for single or paired fish, respectively. (b) The success of each fish on their first disk push in the reversal learning task, where all failures (0) are shown in black, non‐choice trials are shown in gray, and successes (1) are shown in teal or orange for single or paired fish, respectively. [file JFB-107-1106-s006.pdf]

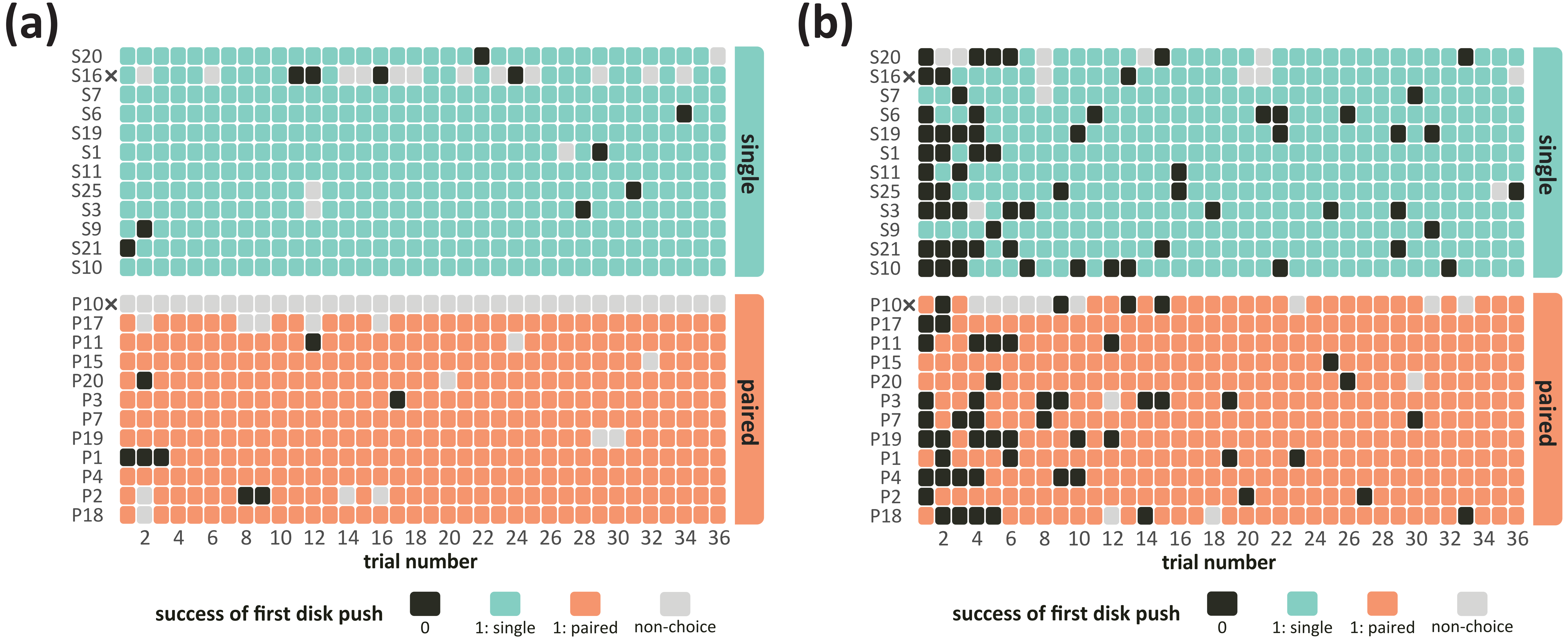

(a)
(b)
S20
S16×
S7
S6
S19
S1
S11
S25
S3
S9
S21
S10
P10×
P17
P11
P15
P20
P3
P7
P19
P1
P4
P2
P18
single
paired
2 4 6 8 10 12 14 16 18 20 22 24 26 28 30 32 34 36
trial number
success of first disk push
0
1: single
1: paired
non-choice
